# Decitabine Augments Chemotherapy-Induced PD-L1 Upregulation for PD-L1 Blockade in Colorectal Cancer

**DOI:** 10.3390/cancers12020462

**Published:** 2020-02-17

**Authors:** Kevin Chih-Yang Huang, Shu-Fen Chiang, William Tzu-Liang Chen, Tsung-Wei Chen, Ching-Han Hu, Pei-Chen Yang, Tao-Wei Ke, K. S. Clifford Chao

**Affiliations:** 1 Translation Research Core, China Medical University Hospital, China Medical University, Taichung 40402, Taiwan; flylerd0425@gmail.com; 2 Department of Nutrition, HungKuang University, Taichung 43302, Taiwan; 3 Lab of Precision Medicine, Feng-Yuan Hospital, Ministry of Health and Welfare, Taichung 42055, Taiwan; suvenchiang@gmail.com; 4 Cancer Center, China Medical University Hospital, China Medical University, Taichung 40402, Taiwan; alice6406@hotmail.com (C.-H.H.); peicheng23@yahoo.com.tw (P.-C.Y.); 5 Department of Colorectal Surgery, China Medical University Hospital, China Medical University, Taichung 40402, Taiwan; wtchen@mail.cmuh.org.tw; 6 Department of Pathology, China Medical University Hospital, China Medical University, Taichung 40402, Taiwan; d14883@mail.cmuh.org.tw; 7 Graduate Institute of Biomedical Science, China Medical University, Taichung 40402, Taiwan

**Keywords:** colorectal cancer, decitabine, microsatellite instability, anti-PD-L1 immunotherapy, tumor microenvironment

## Abstract

Programmed cell death-1 (PD-1) has demonstrated impressive clinical outcomes in several malignancies, but its therapeutic efficacy in the majority of colorectal cancers is still low. Therefore, methods to improve its therapeutic efficacy in colorectal cancer (CRC) patients need further investigation. Here, we demonstrate that immunogenic chemotherapeutic agents trigger the induction of tumor PD-L1 expression in vitro and in vivo, a fact which was validated in metastatic CRC patients who received preoperatively neoadjuvant chemotherapy (neoCT) treatment, suggesting that tumor PD-L1 upregulation by chemotherapeutic regimen is more feasible via PD-1/PD-L1 immunotherapy. However, we found that the epigenetic control of tumor PD-L1 via DNA methyltransferase 1 (DNMT1) significantly influenced the response to chemotherapy. We demonstrate that decitabine (DAC) induces DNA hypomethylation, which not only directly enhances tumor PD-L1 expression but also increases the expression of immune-related genes and intratumoral T cell infiltration in vitro and in vivo. DAC was found to profoundly enhance the therapeutic efficacy of PD-L1 immunotherapy to inhibit tumor growth and prolong survival in vivo. Therefore, it can be seen that DAC remodels the tumor microenvironment to improve the effect of PD-L1 immunotherapy by directly triggering tumor PD-L1 expression and eliciting stronger anti-cancer immune responses, providing potential clinical benefits to CRC patients in the future.

## 1. Introduction

Colorectal cancer (CRC) is one of the leading causes of cancer-related mortality worldwide despite advances in treatment. Until now, combinational chemotherapy FOLFOX regimen [folinic acid, 5-fluorouracil (5-FU), oxaliplatin (OXP)] has been standardized as the first-line treatment for advanced CRC after surgery. However, the appearance of chemoresistance in most patients limits the anti-tumor effect of these combined chemotherapies, leading to tumor escape and distant metastasis within five years [1,2].

Several common cytotoxic agents such as 5-FU and OXP show immunogenic properties that trigger immune responses [3,4,5]. 5-FU remarkably depletes myeloid-derived suppressor cells (MDSCs) within the tumor microenvironment (TME) [6], and OXP triggers immunogenic cell death (ICD) through the surface exposure of calreticulin (CRT), and the release of high mobility group box 1 (HMGB1) and ATP [5,7], which contribute to recruit CD8+ T cell and induce anti-tumor immunity [8]. Moreover, accumulating evidence supports the finding that the intratumoral infiltration of immune cells is associated with a favorable prognosis in CRC [9,10,11]. The clinical efficacies of immunotherapies that target the immune checkpoint programmed cell death 1 receptor (PD-1) and its ligand programmed cell death 1 ligand 1 (PD-L1) have been demonstrated in several malignancies, such as non-small cell lung cancer and bladder cancer [12,13]. However, the majority of CRC patients do not respond to an immune checkpoint blockade (ICB), especially patients with microsatellite-stable (MSS) tumors [14,15,16]. Recent studies have highlighted that intratumoral immune contexts may determine the therapeutic efficacy of the ICB [17,18]. Patients with high intratumoral CD8+T cell infiltration, tumor mutational load, and tumor PD-L1 expression within the TME are more responsive to immunotherapies [19,20]. Therefore, several clinical trials have aimed to reinvigorate T cell immunity to improve the anti-cancer effect of the ICB with combinational therapeutics, such as chemotherapeutic agents combined with radiotherapy, suggesting that desirable ICB efficacy may be expected in CRCs if a suitable immunological TME is reestablished.

However, the function of PD-L1 expression in CRC has not been fully investigated. Our previous studies indicated a strong correlation between tumor PD-L1 expression and CD8+ tumor-infiltrating lymphocytes (TILs), possibly due to the concomitant increase in interferon γ (IFNγ) expression by CD8+ T cells, and the PD-L1 level is positively correlated with favorable prognosis in CRC patients [10,21]. Additionally, the methylation of the *PD-L1* promoter has been shown to negative correlate with its gene expression and is clinically associated with survival, including from prostate cancer, colorectal cancer, acute myeloid leukemia and melanoma [22,23,24,25]. However, the mechanism of the epigenetic regulation of PD-L1 is poorly defined.

In this study, we aimed to provoke an immunogenic microenvironment to upregulate PD-L1 expression by combinational chemotherapy treatment and increase the therapeutic efficacy of anti-PD-L1 immunotherapy. We found that chemotherapeutic drugs directly upregulate tumor PD-L1 expression, and its expression might be modulated by direct epigenetic control. Pharmacologically-induced DNA demethylation or the knockdown of DNA methyltransferase 1 (DNMT1) expression significantly upregulated the tumor PD-L1 level under OXP treatment. Combinational treatment with OXP and an food and drug administration (FDA)-approved DNA demethylation inhibitor (decitabine, DAC) dramatically increased the immunogenicity and PD-L1 expression within the TME in vivo. These results showed that OXP and DAC synergistically enhanced the therapeutic efficacy of anti-PD-L1 immunotherapy in CRC.

## 2. Materials and Methods

### 2.1. Cell Culture and Reagents

The human colorectal cancer cell lines HCT116 and SW480, and mouse colon carcinoma cell line CT26 were cultured in a complete RPMI 1640 growth medium (Thermo Fisher Scientific, CA, USA) with 10% fetal bovine serum (Invitrogen, CA, USA), 3.5 g/L glucose (Thermo Fisher Scientific, CA, USA), 10 mM HEPES (Thermo Fisher Scientific, CA, USA), and 1.0 mM sodium pyruvate (Thermo Fisher Scientific, CA, USA) at 37 °C in an incubator of 5% CO_2_ and 95% air.

The following antibodies were used in this study: anti-PD-L1 (ab205921, clone 28-8, Abcam, Cambridge, UK) anti-PD-L1 (#13684, clone E1L3N, Cell Signaling Technology, MA, USA), anti-β-actin (sc-8432, Santa Cruz, CA, USA), anti-DNMT3a (sc-365769, Santa Cruz, CA, USA), anti-DNMT1 (sc-271729, Santa Cruz, CA, USA), anti-p-signal transducer and activator of transcription 1 (p-STAT1, sc-8394, Santa Cruz, CA, USA), anti-STAT1 (sc-464, Santa Cruz, CA, USA), anti-interferon regulatory factor 1 (IRF1, sc-514544, Santa Cruz, CA, USA), and horseradish peroxidase (HRP)-conjugated secondary antibodies (Santa Cruz, CA, USA).

Lentiviruses carrying individual shRNA were obtained from the National Core Facility for Manipulation of Gene Function by RNAi, miRNA, miRNA sponges, and CRISPR/Genomic Research Center, Academia Sinica, Taipei, Taiwan.

### 2.2. Western Blot Analysis

Total lysates (30 μg) were separated via 6%–12% SDS-PAGE, transferred onto PVDF membranes (Millipore, MA, USA) [26,27], blocked with 5% nonfat milk, incubated with specific antibodies (in 1% non-fat milk) overnight at 4 °C, and probed with HRP-conjugated secondary antibodies. The blot membrane was then incubated with Immobilon Western Chemiluminescent HRP Substrate (Millipore, MA, USA), analyzed by an ImageQuant™ LAS 4000 biomolecular imager (GE Healthcare, Amersham, UK), processed with Adobe Photoshop, and quantified by using ImageJ software (NIH, MD, USA). Each blot was stripped by an immunoblotting stripping buffer (BioLion Tech., Taipei, Taiwan) before incubating with the other antibodies.

### 2.3. Evaluation of the Immunogenic TME Induced by Chemotherapeutic Drugs

**Figure 1:**
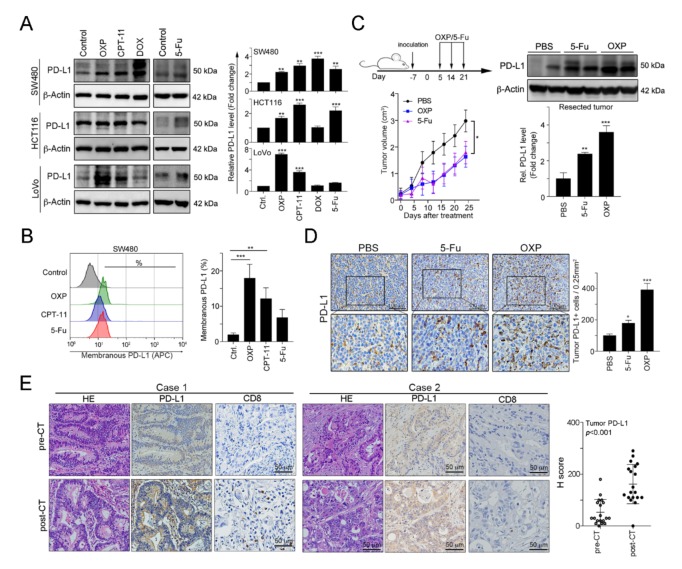
FOLFOX-based preoperative neoadjuvant chemotherapy significantly upregulated the tumor programmed cell death 1 ligand 1 (PD-L1) level. (**A**) The SW480, HCT116 and LoVo colorectal cancer cell lines were treated with oxaliplatin (OXP, 10 μM), irinotecan (CPT-11, 5 mM), doxorubicin (DOX, 1 mM) or 5-fluorouracil (5-Fu, 10 mg/mL) for 24 h and then analyzed by immunoblotting. The quantification of these results is shown (*n* = 3); ** *p* < 0.01 and *** *p* < 0.001. (**B**) SW480 cells were treated with oxaliplatin (OXP, 10 μM), irinotecan (CPT-11, 5 mM) or 5-fluorouracil (5-Fu, 10 mg/mL) for 24 h and then analyzed by flow cytometry. The quantification of these results is shown (*n* = 3); ** *p* < 0.01 and *** *p* < 0.001. (**C**) BALB/c mice were inoculated with CT26 cells (1 × 10^6^ cells/mouse) and then treated with OXP (2.5 mg/kg, intratumoral injection) and 5-Fu (50 mg/kg, intraperitoneal injection) 3 times beginning on day 5, with 7-day intervals between injections. The tumors were resected from representative mice, and then they were homogenized and then analyzed by immunoblotting. The quantification of these results is shown (*n* = 3); ** *p* < 0.01 and *** *p* < 0.001. (**D**) The resected tumors from representative mice were stained for PD-L1 by immunohistochemistry. The quantification of these results is shown (*n* = 3); * *p* < 0.05 and *** *p* < 0.001. (**E**) Tumor samples from metastatic colorectal cancer patients (*n* = 19) were harvested before and after the patients received FOLFOX-based neoadjuvant chemotherapy (neoCT). Representative immunohistochemical images show the results from the patients’ pre-neoCT tumor biopsy and post-neoCT surgical tissue sample. Each parameter for the individual patients is shown. The histoscores (H-scores) for tumor PD-L1 expression indicated that neoCT impacts tumor PD-L1 expression (*n* = 19, Wilcoxon matched-pairs test, *p* < 0.001). Case 1: PD-L1 level was upregulated in response to FOLFOX regimen; Case 2: PD-L1 level was unresponsive to FOLFOX regimen.

Six-week-old female BALB/c mice were administrated according to the institutional guidelines approved by Institutional Animal Care and Use Committee of China Medical University [Protocol No.: CMUIACUC-2018-167]. Briefly, CT26 cells (1 × 10^6^ cells/mouse) were suspended in 100 μL of Matrigel, and they were then subcutaneously inoculated into the right flank of the mouse. After 7 days, oxaliplatin (2.5 mg/kg/mouse, intratumoral injection) and 5-Fu (50 mg/kg/mouse, intraperitoneal injection) were administered 3 times with 7-day intervals between injections (Figure 1C). The tumor volume was measured and recorded every 3 days throughout the study. For the combination treatment of decitabine (DAC) and OXP, 6-week-old female BALB/c mice were subcutaneously inoculated with CT26 cells (5 × 10^5^ cells/mouse) that were suspended in 100 μL 50% Matrigel in the right flank. After 7 days, oxaliplatin (6 mg/kg/mouse, intraperitoneal injection) was administered 4 times with 3-day intervals between injections, and decitabine (20 μg/mouse) was administered for 5 consecutive days. The tumor volume was measured every 3 days and then calculated by the formula: V = (L × W^2^)/2. [L: longest diameter; W: shortest diameter; and V: volume]. The mice were sacrificed at the end of the experiments for further analysis by immunoblotting and immunohistochemistry.

### 2.4. Patient Tissue Specimens and Clinicopathological Characteristics and the Construction of a Tissue Microarray (TMA)

The patient tissue samples used in our study were approved by the Institutional Research Ethics Committee at the China Medical University Hospital on 21 March 2018 [Protocol number: CMUH107-REC2-008], and the patients were informed before the use of their clinical material. Nineteen patients were histologically and clinically diagnosed in the China Medical University Hospital between 2006 and 2014. The patients received FOLFOX-based preoperative neoadjuvant chemotherapy (neoCT) and then surgery. The collected tissue specimens conformed to the criteria: >70% tumor cells in tumor tissue (percentage of) and a corresponding normal mucosal tissue with distal region (>5 cm from the edge of tumor region) [28]. Tissue microarrays (TMA) were constructed from 19 pre-neoCT biopsies and post-neoCT resected surgical specimens [10]. A maximum of 60 punches were placed in a single block with 2 mm diameter of each cylinder for embedding and sliding [29,30].

### 2.5. Detection of the Surface PD-L1 Level on Tumor Cells

SW480, HCT116 and CT26 cells were treated with the indicated chemotherapeutic drugs for 24 h, and then the cells were collected with a dissociation buffer (Thermo Fisher Scientific, CA, USA). The cells were blocked with 5% bovine serum albumin (BSA, Invitrogen, CA, USA) and then stained with an allophycocyanin (APC)-conjugated anti-PD-L1 antibody (clone 29E.2A3, BioLegend, CA, USA). All events were acquired by a BD LSR-II flow cytometer (BD Biosciences, Mountain View, CA, USA), and data were processed with the FlowJo software (TreeStar, Ashland, Oregon, USA).

### 2.6. Immunohistochemistry (IHC)

The antibodies used for immunohistochemistry (IHC) were as follows: anti-PD-L1 (Abcam, Cambridge, UK), anti-mouse CD3 (BioLegend, CA, USA), anti-mouse CD8a (BioLegend, CA, USA) and anti-mouse CD86 (BioLegend, CA, USA). Slides (3 µm thickness) were stained with the HRP-conjugated Vectastain Elite ABC Kit (Vector Laboratories, CA, USA) according to the manufacture’s protocol, incubated with DAB chromogen (Vector Laboratories) and counterstained by hematoxylin. The PD-L1 staining patterns were evaluated and scored based on the intensity and percentage of positive cells for histoscore (H-score), which was calculated by a semiquantitative assessment of both the intensity of the staining (0: negative staining; 1: weak; 2: moderate; and 3: strong) and the percentage of immunopositive cells. The range of the H-score was from 0 to 300. The PD-L1 expression status was categorized as low or high according to the median value of the H-score. Staining for CD3 and CD8a was positive when detected in the intratumor-infiltrating lymphocytes (TILs) and was evaluated by using a microscope under 400× magnification (no. of TILs/400X magnification) for evaluation [9].

### 2.7. RNA Sequencing (RNA-Seq) and Data Analysis

Total RNA was extracted from the resected tumors of the PBS, OXP, DAC, and DAC plus OXP groups on day 5 (1 day after completing DAC treatment) by using the TRIzol reagent. Message RNA was extracted from the total RNA and cut into short fragments with ~200 bases as templates for cDNA synthesis. The cDNAs subsequently used to establish a cDNA library by PCR amplification and sequenced by using an Illumina HiSeqTM 2500 platform [31]. Clean reads were obtained by trimming the adaptor sequences from raw reads, and these reads were then were used for further transcript annotation and calculation bases on the fragments per kilobase per million reads (FPKM) method. Differential gene expressions (DEGs) were identified with the DESeq software package. The Benjamini–Hochberg false discovery rate was employed to correct the *p* values with a significant level set at 0.05 [31].

### 2.8. Administration of Mice with DAC Followed by Chemotherapy and PD-L1 Immunotherapy

A total of 5 × 10^5^ CT26 cells in 100 μL of 50% Matrigel were inoculated into the right flank of the BALB/c mice. The treatments were initiated on day 7 after tumor inoculation: DAC (intraperitoneal injection, 10 μg/mouse for 3 consecutive days) and OXP (intraperitoneal injection, 2.5 mg/kg/mouse, 5 times with 3-day intervals between administrations). On day 11, a PD-L1 blockade was performed (100 μg/mouse, intraperitoneal injection, 4 times with 3-day intervals between administrations, Bio×Cell clone 10F.9G2, NH, USA). Tumor volume (V) was calculated by the formula: V = (L × W^2^)/2 every 3 days. The mice were sacrificed when the longest diameter reached 20 mm, and the survival of the tumor-bearing mice was recorded every 3 days.

### 2.9. Statistical Analysis

All experiments were carried out at least 3 times. The statistical analysis was performed by using the GraphPad Prism 7 statistical software (San Diego, CA, USA) with a two-way ANOVA followed by Bonferroni’s post hoc test, a one-way ANOVA followed by Dunnett’s post hoc test, or an unpaired *t*-test where appropriate. Data are expressed as mean +SD. A Student’s *t*-test was used to compare the differences between two groups. A Kaplan–Meier survival analysis and the log-rank test were used to compare the survival rates of the mice. *p* < 0.05 was considered to indicate a statistically significant difference.

## 3. Results

### 3.1. Preoperative Neoadjuvant Chemotherapy Stimulated Tumor PD-L1 Expression within Tumors in Colorectal Cancer

Previous studies have demonstrated that DNA damaging agents elicit tumor PD-L1 upregulation, especially double-stranded DNA (dsDNA) damaging drugs [32]. To evaluate the influences of chemotherapeutic drugs on tumor PD-L1 expression, we treated human colorectal cancer cell lines with these agents, which are extensively used in CRC patients. Doxorubicin (DOX), the topoisomerase II inhibitor that is the dsDNA break inducer, was used as positive control for tumor PD-L1 upregulation [32]. We found that all first-line chemotherapeutic drugs that are used in colorectal cancer, specifically 5-FU, OXP and irinotecan (CPT-11), provoked tumor PD-L1 expression upregulation to different extents (Figure 1A). Among these drugs, the topoisomerase I inhibitor CPT-11 and, especially, the alkylating agent OXP significantly increased tumor PD-L1 levels and surface PD-L1 expression (Figure 1B). To confirm the effect of OXP on tumor PD-L1 expression in vivo, we analyzed resected tumors from the BALB/c mice that received an OXP-based or 5-Fu-based chemotherapy regimen (Figure 1C). We found that both 5-Fu and OXP remarkably increased the diverse extent of tumor PD-L1 expression. However, OXP was more profoundly involved in tumor PD-L1 expression upregulation and membrane translocation (Figure 1C,D), suggesting that chemotherapeutic drugs efficiently mediated tumor PD-L1 expression in vitro and in vivo.

To address the immunologic effect of chemotherapeutic regimen on the TME in colorectal cancer patients, we analyzed 19 tumor tissue samples from patients with stage IV metastatic CRC before and after received a neoadjuvant FOLFOX regimen (folinic acid, 5-FU and OXP: pre-neoCT biopsies and post-neoCT surgical tissue samples). The patients’ characteristics are summarized in Table 1. Based on the results of immunohistochemical analysis, our results showed that tumor PD-L1 expression was significantly higher in the tissue samples after chemotherapy (pre-neoCT median H-score: 30; post-neoCT median H-score: 130, *p* < 0.001, Figure 1E, case 1). Interestingly, FOLFOX chemotherapy was associated with an increase of CD8+ TILs (Figure 1E, case 1). Taken together, these results support the conclusion that chemotherapy-induced CD8+ TIL recruitment results in the release of IFNγ, which upregulates PD-L1 expression, confirming that OXP-based preoperatively neoadjuvant chemotherapy may provoke immunologic status for the application of immunotherapy in CRC patients.

### 3.2. Pharmacologically-Induced DNA Demethylation Directly Triggered the Upregulation of Tumor PD-L1 Expression

However, we found that tumor PD-L1 expression was not upregulated by preoperative FOLFOX chemotherapy in a population of stage IV metastatic CRC patients (post-surgical specimens: tumor PD-L1^H^ (Figure 1E, case 1) vs. tumor PD-L1^L^ (Figure 1E, case 2): 63% vs. 37%). Similarly, our previous study indicated that the tumor PD-L1 level was not upregulated by preoperatively neoadjuvant chemoradiotherapy (neoCRT) in 36% of locally advanced rectal cancer (LARC) patients (tumor PD-L1^H^ vs. tumor PD-L1^L^: 64% vs. 36%) [11,21], suggesting that other mechanisms may influence the PD-L1 induction.

**Figure 2:**
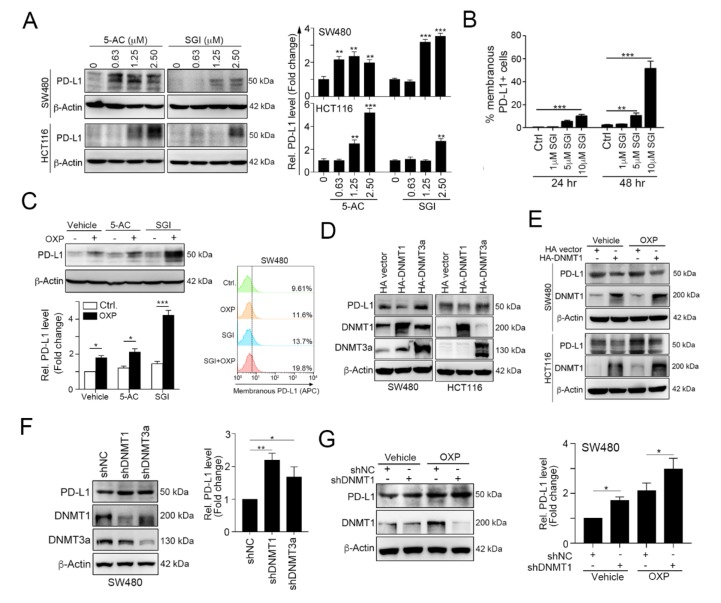
Epigenetic modifications mediated by DNA methyltransferase 1 (DNMT1) directly inhibits tumor PD-L1 expression. (**A**) SW480 and HCT116 cells were treated with the DNA hypomethylation inducers 5-azacytidine (5-AC) and SGI-1027 (SGI) for 24 h and then analyzed by immunoblotting. The quantification of these results is shown on the right (*n* = 3); ** *p* < 0.01 and *** *p* < 0.001. (**B**) SW480 cells were treated with various concentrations of SGI-1027 for 24 and 48 h and then analyzed by flow cytometry. The quantification of these results is shown on the right (*n* = 3); ** *p* < 0.01 and *** *p* < 0.001. (**C**) SW480 were simultaneously treated with OXP (10 μM), 5-AC (2.5 μM) and SGI (2.5 μM) for 24 h and then analyzed by immunoblotting. SW480 cells were simultaneously treated with OXP (10 μM) and SGI (2.5 μM) for 24 h and then analyzed by flow cytometry. (**D**) SW480 and HCT116 cells were separately transfected with hemagglutinin (HA)-DNMT1 and HA-DNMT3a for 48 h and then analyzed by immunoblotting. The quantification of these results is shown in Figure S1. (**E**) SW480 and HCT116 cells were separately transfected with HA-DNMT1 for 24 h and then treated with OXP (10 μM) for 24 h. Cell lysates were harvested and examined by immunoblotting. (**F**) SW480 cells were infected with a lentivirus carrying a negative control (NC), DNMT1 or DNMT3a shRNA. After selecting stable knockdown cells by puromycin selection, the cells were harvested and then analyzed by immunoblotting. The quantification of these results is shown on the right (*n* = 3); * *p* < 0.05 and ** *p* < 0.01. (**G**) SW480-shNC and SW480-shDNMT1 cells were treated with OXP (10 μM) for 24 h. Cell lysates were harvested and then analyzed by immunoblotting. The quantification of these results is shown on the right (*n* = 3); * *p* < 0.05.

Previous studies have indicated that the DNA methylation of the *PD-L1* promoter is associated with PD-L1 expression [22,23]. Therefore, we aimed to evaluate the effect of DNA methyltransferase (DNMT) inhibitors 5′-azacitidine (5-AC) and SGI-1027 (SGI) on PD-L1 expression. As shown in Figure 2A, we found that PD-L1 level gradually increased according to the dose of the DNMT inhibitor in colorectal cancer (Figure 2A). The surface expression of tumor PD-L1 was also significantly upregulated by the DNMT inhibitor SGI-1027 (Figure 2B). Moreover, DNMT inhibitors synergistically enhanced OXP-induced PD-L1 expression (Figure 2C) and membranous PD-L1 protein expression (Figure 2C).

To investigate which DNMT protein participated in PD-L1 regulation, we overexpressed DNMT1 and DNMT3a, and then we examined the expression of PD-L1 (Figure 2D and Figure S1). These results showed that the overexpression of DNMT1 clearly reduced PD-L1 expression, but DNMT3a did not have a significant effect on the PD-L1 level. Furthermore, the overexpression of DNMT1 dramatically alleviated OXP-induced PD-L1 expression (Figure 2E). In contrast, knocking down DNMT1 expression significantly increased PD-L1 expression (Figure 2F) and enhanced OXP-induced PD-L1 expression (Figure 2G). Taken together, these results suggest that the tumor PD-L1 level is directly regulated by DNMT1.

**Figure 3:**
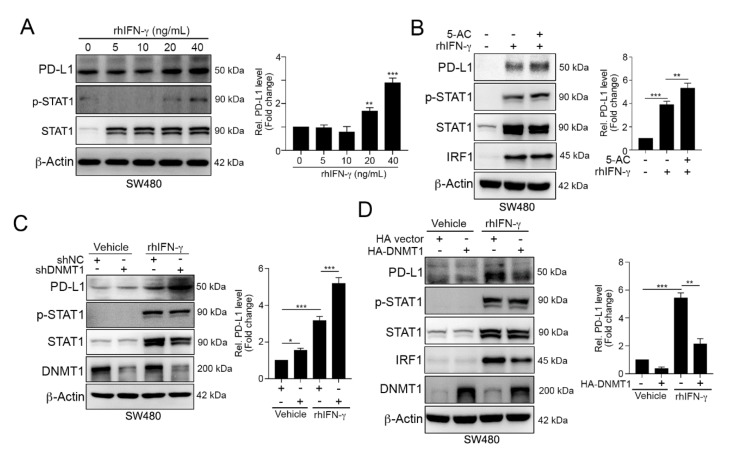
DNMT1 influenced the IFNγ-mediated upregulation of PD-L1 expression. (**A**) SW480 cells were treated with different concentrations of recombinant human IFN-γ (rhIFN-γ) for 24 h and then analyzed by immunoblotting. The quantification of these results is shown on the right (*n* = 3); ** *p* < 0.01 and *** *p* < 0.001. (**B**) SW480 cells were cotreated with rhIFN-γ (10 ng/mL) and 5-AC (2.5 μM) for 24 h and then examined by immunoblotting. The quantification of these results is shown on the right (*n* = 3); ** *p* < 0.01 and *** *p* < 0.001. (**C**) SW480-shNC and SW480-shDNMT1 cells were directly treated with IFN-γ (10 ng/mL) for 24 h and then analyzed by immunoblotting. The quantification of these results is shown on the right (*n* = 3); * *p* < 0.05 and *** *p* < 0.001. (**D**) SW480 cells were transfected with HA-vector or HA-DNMT1 for 24 h and then treated with a high-dose of IFN-γ (40 ng/mL) for 24 h. Cell lysates were harvested and then analyzed by immunoblotting. The quantification of these results is shown on the right (*n* = 3); ** *p* < 0.01 and *** *p* < 0.001. These data were obtained from three independent experiments, and the values represent the mean ± S.D.

Our previous studies indicated that chemoradiotherapy-damaged tumor cells release IFNγ, which remarkably promotes the upregulation of PD-L1 expression [21]. Indeed, we found that the administration of IFNγ resulted in STAT1 phosphorylation and directly increased tumor PD-L1 expression (Figure 3A). Moreover, the effect of IFNγ on PD-L1 upregulation was dramatically increased when cells were simultaneously treated with 5-AC (Figure 3B). The suppression of DNMT1 by lentivirus-carrying shRNA against DNMT1 also significantly increased the effect of IFNγ on PD-L1 upregulation (Figure 3C). Conversely, the overexpression of DNMT1 clearly diminished IFNγ-induced PD-L1 upregulation (Figure 3D). These results indicated that *PD-L1* promoter methylation by DNMT1 may decrease IFNγ-induced PD-L1 upregulation.

**Figure 4:**
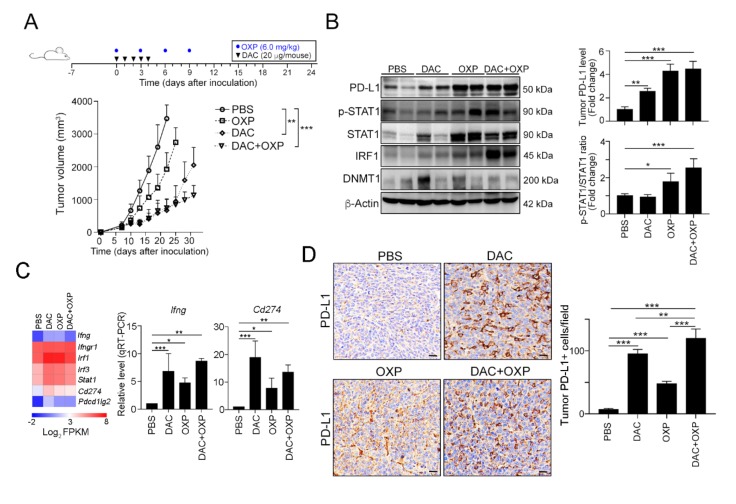
Decitabine synergistically increased the therapeutic response to immunogenic chemotherapy in vivo. (**A**) Tumor-bearing BALB/c mice (*n* = 6) were treated with OXP (6 mg/kg, i.p. injection) and decitabine (DAC, 20 μg/mouse, i.p. injection) at the indicated times shown on the timeline. Tumor volume was measured and calculated every 3 days. The quantification of these results is shown (*n* = 6); ** *p* < 0.01 and *** *p* < 0.001. (**B**) Resected tumors from representative mice were collected for lysis and then analyzed by immunoblotting. The quantification of these results is shown (*n* = 3); * *p* < 0.05, ** *p* < 0.01 and *** *p* < 0.01. (**C**) After treatment with DAC for 5 consecutive days, tumors were resected from representative mice, analyzed by RNA-seq (*n* = 2), and validated by qRT-PCR (*n* = 3). (**D**) Resected tumors from representative mice were analyzed by immunohistochemistry. Bar: 10 μm. The quantification of these results is shown (*n* = 4); ** *p* < 0.01 and *** *p* < 0.01.

To evaluate the therapeutic effects of the combination of OXP and DNA methyltransferase inhibitors (DNMTi), we inoculated BALB/c mice with CT26 cells and treated the mice with the FDA-approved small molecular DNMT inhibitor 5-aza-2′-deoxycytidine (decitabine, DAC, 20 μg/mouse by intraperitoneal injection, i.p. injection) and OXP (6 mg/kg by i.p. injection). There was no significant body weight loss in the DAC and OXP individual groups, but 5%–10% body weight reduction was observed in the DAC and OXP group. As shown in Figure 4A, OXP alone slightly decreased tumor growth (Dunnett’s test, *p* = 0.046), but DAC significantly decreased tumor growth (Dunnett’s test, *p* = 0.01). A profound effect was observed in the DAC and OXP group (Dunnett’s test, *p* < 0.01, Figure 4A). Moreover, the results of immunoblotting showed that the tumor PD-L1 protein level was highly increased, and the phosphorylation of STAT1 and its downstream transcription factor IRF1 was significantly increased in the resected tumors from the DAC and OXP group (Figure 4B). These results show that DNMT1 epigenetically controlled PD-L1 expression in CRC cells in vivo.

In addition to the direct impact on PD-L1 expression, DAC has been reported to remodulate the TME for immunogenicity [33], we conducted an RNA-seq analysis on the resected tumors from these groups (Figure 4C and Figure S2). RNA-seq results suggested that the mRNA expression profile was profoundly altered (Figure S2A). Lots of functional immune-related genes were differentially expressed under DAC administration. Compared to the PBS group, the DAC group included more than 149 significantly upregulated genes (red) and 15 significantly downregulated genes (Figure S2B). A similar gene profile was observed in the DAC and OXP group, with 115 genes exhibiting a significantly upregulated expression (red) and 26 genes exhibiting a significantly downregulated expression (Figure S2C).

Furthermore, the levels of *Ifng*, *Ifngr1*, *Irf1*, *Irf3*, *stat1*, *Cd274 (Pdl1)* and *Pdcd1lg2 (Pdl2)* were dramatically upregulated in the DAC-treated group and highly expressed in the DAC and OXP-treated group (Figure 4C). The expression of *Ifng* and *Cd274 (Pdl1)* was validated by qRT-PCR. The level of *Cd274 (Pdl1)* was significantly increased in the DAC-treated group and the DAC and OXP-treated group. Similarly, an immunohistochemical analysis showed that the level and distribution of PD-L1 was strongly altered in the DAC-treated group and widely increased in the DAC and OXP-treated group (Figure 4D). These results implied that the DNMTi may remodel the TME to promote PD-L1 expression in vivo.

### 3.3. Decitabine Provokes Immune Signatures for Antitumor Immunity

**Figure 5:**
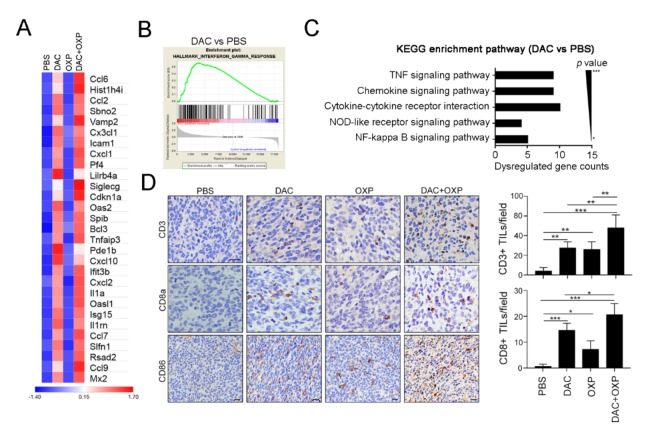
Decitabine remodeled the immunogenicity of the tumor microenvironment in colorectal cancer (CRC) in vivo. (**A**) Resected tumors from representative mice were analyzed by RNA-seq (*n* = 2), and the expression of immune-related genes was significantly upregulated by DAC. (**B**) The gene set enrichment analysis (GSEA) plot for the signature “interferon-γ response,” which represents a set of IFN-γ-related genes that exhibited an upregulated expression after treatment with DAC for 5 consecutive days, is shown. (**C**) The immune-related gene numbers were plotted based on KEGG classification, and the *p* value of each category. (**D**) Resected tumors from representative mice were analyzed for CD3+ intratumoral-infiltrating lymphocytes (TILs), CD8a+ TILs, and CD86 expression by immunohistochemistry (*n* = 4). Bar: 10 μm. The quantification of these results is shown (*n* = 4); * *p* < 0.01, ** *p* < 0.01 and *** *p* < 0.01.

Furthermore, we found that cytokine-associated genes (*Tnfaip3*, *Ifit3b*, *Il1a*, *Isg15*, *Il1rn*, *Mx2*), chemokine-associated genes (*Ccl6*, *Ccl2*, *Cx3cl1*, *Cxcl1*, *Cxcl10*, *Cxcl2*, *Ccl7*, *Cxcl9*), STAT signaling, and immune response genes (*Sbno2*, *Icam1*, *Pf4*, *Lilrb4a*, *Spib*, *Rsad2*) were activated after DAC exposure (Figure 5A). Notably, the genes related to the immune response exhibited a significantly upregulated expression (Figure 5A), which suggested that tumor immunogenicity may be improved by DAC.

To investigate potentially overactivated pathways, we performed a gene set enrichment analysis (GSEA) to compare the gene profiles between the untreated and DAC-treated groups. The GSEA of the untreated and DAC-treated groups revealed that the most enriched pathways were the IFNγ response (Figure 5B), the IFNα response (Figure S3A), Janus kinase (JAK)/STAT signaling (Figure S3B), and the inflammatory response (Figure S3C). Similar results were observed in the DAC and OXP group, and the GSEA results showed the gene profiles for IFNγ response (Figure S4A), the IFNα response (Figure S4B), JAK/STAT signaling (Figure S4C), and the inflammatory response (Figure S4D) were significantly upregulated. In comparison with those of the untreated group, the affected genes in the DAC-treated group were analyzed by Kyoto Encuclopedia of Genes and Genomes (KEGG) gene ontology analysis (Figure 5C). Significantly dysregulated immune-related genes, such as the tumor necrosis factor (TNF) signaling pathway, the chemokine signaling pathway, and the cytokine receptor pathway, were observed. These results suggested that DAC treatment may modify the transcriptomic profiles of immune activation and the interferon signaling pathway genes to remodel the TME, and this remodeling may recruit immune cells such as T cells for antitumor immunity. Indeed, the results of an immunohistochemical analysis showed that increasing numbers of CD3+ and CD8a+ TILs infiltrated into the tumor area after DAC treatment (Figure 5D). More intratumoral CD3+ and CD8a+ TILs were observed in the DAC and OXP group than in any other group. The expression of the immunogenicity marker CD86 was also increased in the DAC-treated group and the DAC and OXP-treated group, suggesting that DAC may provoke tumor immunogenicity in vivo.

### 3.4. Improvement of the Therapeutic Effect of PD-L1 Immunotherapy with DAC and OXP

**Figure 6:**
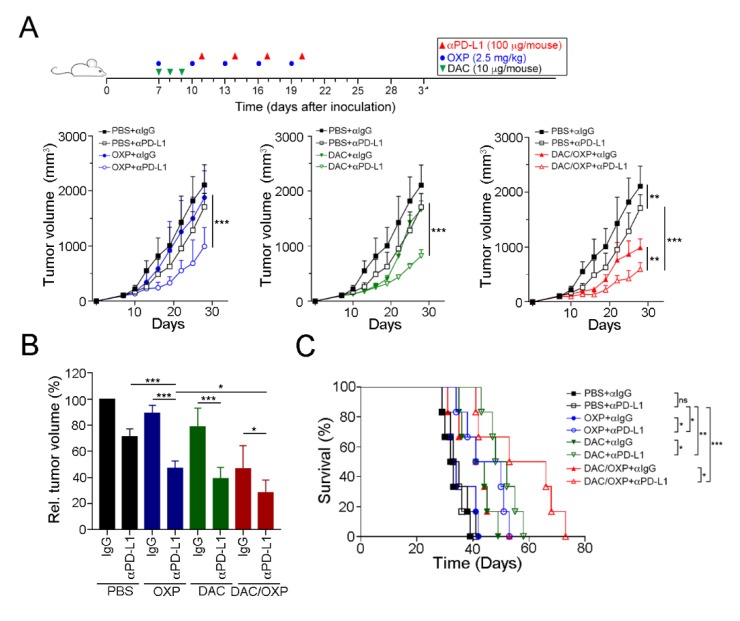
Decitabine enhanced the therapeutic efficacy of anti-PD-L1 therapy in CRC in vivo. (**A**) Tumor-bearing BALB/c mice (*n* = 6) were treated with OXP (2.5 mg/kg, i.p. injection), decitabine (DAC, 10 μg/mouse, i.p. injection) and the PD-L1 blockade (100 μg/mouse, i.p. injection) at the indicated times shown on the timeline. Tumor volume was measured and calculated every 3 days. The results of the OXP and DAC alone groups are displayed in Figure S3. The quantification of these results is shown (*n* = 6); ** *p* < 0.01 and *** *p* < 0.001. (**B**) Relative tumor volume was calculated. The quantification of these results is shown (*n* = 6); * *p* < 0.05 and *** *p* < 0.001. (**C**) Mouse survival was assessed with Kaplan–Meier survival curves (*n* = 6); * *p* < 0.05, *p* < 0.01 and *** *p* < 0.001.

Since DAC could remodel TME immunogenicity to trigger T lymphocyte infiltration and directly upregulate PD-L1 expression, we assessed the therapeutic response to a PD-L1 blockade (100 μg/mouse by i.p. injection) with the additions of DAC (10 μg/mouse by i.p. injection) and OXP (2.5 mg/kg by i.p. injection) (Figure 6A). BALB/c mice who had tumors were administered with various drug combinations. The results showed that OXP with the PD-L1 blockade (*n* = 6) had a stronger effect on tumor growth inhibition compared to PD-L1 blockade alone (Tukey *t*-test, *p* < 0.001, Figure 6A). The relative tumor volume was significantly decreased (71.2% ± 6.0% vs. 47.1% ± 5.6%, respectively, *p* = 0.0032, Figure 6B), and the survival rate was improved (Figure 6C, median survival in days: 34 vs. 45.5 days, respectively, log-rank test, *p* = 0.0207). DAC with the PD-L1 blockade (*n* = 6) also had a significant effect on tumor growth compared to PBS with the PD-L1 blockade (Tukey *t*-test, *p* < 0.001, Figure 6A). The relative tumor volume was profoundly decreased (71.2% ± 6.0% vs. 39.1% ± 8.6%, respectively, *p* < 0.001, Figure 6A), and survival times were prolonged (Figure 6C, median survival in days: 34 vs. 50 days, respectively, log-rank test, *p* < 0.001) in the DAC with the PD-L1 blockade group compared to the PBS with the PD-L1 blockade group. Moreover, the combinational treatment of DAC and OXP with the PD-L1 blockade showed a larger inhibitory effect on tumor growth (Tukey’s *t*-test, *p* < 0.001, Figure 6A) and produced smaller tumor volumes (71.2% ± 6.0% vs. 23.8% ± 7.8%, respectively, *p* < 0.001, Figure 6B) and longer survival times (median survival in days: 34 vs. 59.5 days, respectively, log-rank test, *p* < 0.001, Figure 6C) than PBS with the PD-L1 blockade. Therefore, our results suggested that DAC not only modified the immunogenic TME to recruit more T lymphocytes but also directly provoked tumor PD-L1 expression, creating a more accessible target for the PD-L1 blockade. Taken together, DAC enhanced the therapeutic efficacy of the PD-L1 blockade and improved the survival rate in CRC.

## 4. Discussion

In this study, we found that the epigenetic modification on *PD-L1* promoter by DNMT1 influenced PD-L1 expression. Moreover, the inhibition of DNMT1 activity not only upregulated the PD-L1 level but also remodeled the immunogenicity within the tumor microenvironment in vitro and in vivo. DAC combined with immunogenic chemotherapy profoundly enhanced the therapeutic efficacy of anti-PD-L1 immunotherapy in colorectal cancer. Taken together, our results showed that TME remodulation by DAC profoundly enhanced PD-L1 expression for targeting by the PD-L1 blockade.

Anti-PD1/PD-L1 immunotherapies have achieved exciting clinical outcomes in several malignancies, such as non-small cell lung cancer (NSCLC), bladder cancer and melanoma. However, the overall response rate of PD-1/PD-L1 immunotherapies in solid tumors is less than 30% because the inhibitory status within the TME largely impairs therapeutic efficacy. The intensity of the immune response within the TME such as the density of TILs, the PD-L1 status, and the microsatellite instability (MSI) profoundly determines the efficiency of immunotherapy. Our previous studies demonstrated that preoperative chemoradiotherapy (neoCRT) promotes CD8+ TIL infiltration for IFNγ secretion, driving PD-L1 upregulation in tumor cells [9,10,21]. Consistent with our previous studies, this study indicated that both 5-FU and OXP, the first-line chemotherapeutic drugs for CRC, directly triggered tumor PD-L1 upregulation in vitro and in vivo. Similar phenomena of tumor PD-L1 expression upregulation were observed in metastatic CRC patients who preoperatively received a FOLFOX-based (Folic acid, 5-FU and OXP) chemotherapy regimen. These results are consistent with the adaptive immune resistance, which is defined as a compensatory mechanism of tumor escape within the TME [13,34,35,36]. The upregulation of tumor PD-L1 in response to IFNγ from CD8 T cells is an adaptive immune resistance mechanism that similarly occurs in response to FOLFOX- and Xeloda-based chemotherapy [34,36]. This finding could represent an adaptive immune resistance mechanism to escape the impact of chemotherapy via tumor PD-L1 upregulation, which could be easily targeted by the PD1/PD-L1 blockade.

More attractively, our results showed that few patients displayed a low tumor PD-L1 level within the TME even after neoadjuvant chemotherapy treatment. Therefore, remodeling low immunoreactivity (cold tumor) into high immunoreactivity (hot tumor) within the TME has become more important for therapeutic strategies, such as dendritic cell (DC) vaccines and radiotherapy. Recent findings have shown that hypermethylation of the *PD-L1* promoter is associated with poor overall survival (OS) and recurrence-free survival (RFS) and can be considered an independent prognostic factor in several malignances such as colorectal cancer, prostate cancer, acute myeloid leukemia (AML) and melanoma [22,23,24,25]. In addition, the expression of PD-L1 is regulated by DNA methylation in response to transforming growth factor-β (TGF-β) or nuclear factor kappa B (NF-kB) signaling in non-small cell lung cancer [37]. Consistent with these findings, we found that the hypermethylation of the *PD-L1* promoter in colorectal cancer was associated with a decreased PD-L1 expression and influenced the response to IFNγ signaling. Moreover, we demonstrated that PD-L1 expression could be altered in human colorectal cancer by using DNA hypomethylating agents, providing more information about enhancing the response to the PD1/PD-L1 blockade.

Furthermore, our results showed that DNA hypomethylating agents can modify immune profiles by demethylating DNA promoter regions, and this effect can increase the expression of immune-associated genes, such as cytokine genes, and activate chemokine gene signatures. These results clearly indicated that the intensity of the immune response within the TME was changed by DAC. Moreover, increasing evidence has shown that DNA hypomethylation inducers can remarkably improve the expression of tumor-associated antigens (TAA) in several malignancies [38,39,40]. Our findings also revealed that DAC could revoke and reactivate tumor-related genes, boosting host-tumor immune responses and promoting the intratumoral infiltration of T cells in vivo [41]. These results implied that the FDA-approved DNA hypomethylation inducers 5′-azacitidine and decitabine may be the ideal pretreatment drugs for immunotherapy [42]. Consistent with our results, Yu et al. demonstrated that low-dose decitabine administration improves the immunogenicity within the TME in vivo [33]. Furthermore, Chiappinelli et al. also reported that DNMTi directly trigger the type I interferon response, which sensitizes melanoma to anti-cytotoxic T-lymphocyte-associated protein 4 (CTLA4) immunotherapy [43]. Similarly, our results showed that DAC altered the molecular characteristics of cancer cells so that the cells became more immunoresponsive, resulting in the remodeling of the TME and increasing the infiltration of immune cells into the tumor site for the immunomodulation of the tumor. Moreover, our colorectal cancer animal model showed that a DNA hypomethylating agent has the potential to alter the immune profiles in MSS CRC (CT26 cells have been proven to be MSS [33,36]). In addition, the combined DAC and PD-L1 blockade profoundly delayed CT26 tumor growth and prolonged the survival rate. Our findings suggest that the therapeutic strategy of the combined DAC and PD-L1 blockade would be effective in metastatic CRC.

## 5. Conclusions

Collectively, we have provided promising therapeutic strategies for boosting the low response rate to the PD1/PD-L1 blockade in CRC patients. DAC remodeling the TME could create more feasible conditions for anti-PD-L1 immunotherapies, especially for MSS-CRC patients. However, further studies are needed to determine whether the methylation of the PD-L1 promoter influences the survival outcomes of CRC patients who are treated with the PD-L1 blockade.

## Figures and Tables

**Table 1:** Characteristics of patients with colorectal cancer (*n* = 19).

Clinicopathological Parameters	Total No. (%)	Pre-NeoCT		Post-NeoCT	
		High	Low	High	Low
	19	2	17	12	7
Age					
>65	7	1	6	4	3
<65	12	1	11	8	4
Sex					
Female	5	1	4	2	3
Male	14	1	13	10	4
Primary tumor location					
Colon	13	2	11	9	4
Rectum	6	0	6	3	3
Lymphovascular invasion (LVI)					
Present	11	1	10	7	4
Absent	8	1	7	5	3
Perineural Invasion (PNI)					
Present	15	1	14	8	7
Absent	4	1	3	4	0

Expression level of each component was categorized as low or high according to the median value of the H-score.

## Supplementary Materials

The following are available online at https://www.mdpi.com/2072-6694/12/2/462/s1.
