# Supplementary material for: Decitabine Augments Chemotherapy-Induced PD-L1 Upregulation for PD-L1 Blockade in Colorectal Cancer
Source: Cancers (Basel). 2020 Feb 17;12(2):462. doi: 10.3390/cancers12020462 (PMC7072566; doi:10.3390/cancers12020462)
Supplement: Supplementary file 1 [file cancers-12-00462-s001.pdf]

Supplementary information

Decitabine augments chemotherapy-induced PD-L1 upregulation for PD-L1 blockade in colorectal cancer

Kevin Chih-Yang Huang[1,2], Shu-Fen Chiang[3,4], William Tzu-Liang Chen[5], Tsung-Wei Chen[6,7], Ching-Han Hu[4], Pei-Chen Yang[4], Tao-Wei Ke[5,*] and K. S. Clifford Chao[4,*]

[1] Translation Research Core, China Medical University Hospital, China Medical University, Taichung 40402, Taiwan
[2] Department of Nutrition, HungKuang University, Taichung 43302, Taiwan
[3] Lab of Precision Medicine, Feng-Yuan Hospital, Taichung 42055, Taiwan
[4] Cancer Center, China Medical University Hospital, China Medical University, Taichung 40402, Taiwan
[5] Department of Colorectal Surgery, China Medical University Hospital, China Medical University, Taichung 40402, Taiwan
[6] Department of Pathology, China Medical University Hospital, China Medical University, Taichung 40402, Taiwan
[7] Graduate Institute of Biomedical Science, China Medical University, Taichung 40402, Taiwan

Tao-Wei Ke and K. S. Clifford Chao contributed equally.
Corresponding author: K. S. Clifford Chao
Cancer Center, China Medical University Hospital, China Medical University, Taichung 40402, Taiwan
E-mail: d94032@mail.cmuh.org.tw
Tel.: 886-4-22052121 ext. 2976
Fax: 886-4-22075011

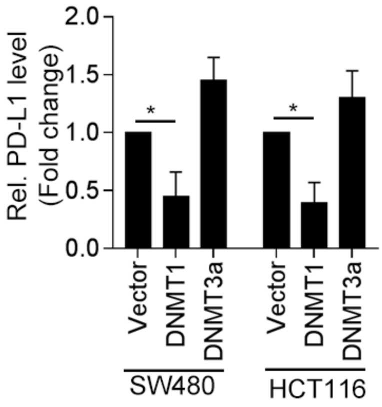

Figure S1. Overexpression of DNMT1 significantly reduced tumor PD-L1 expression

SW480 and HCT116 cells were separately transfected with HA-DNMT1 and HA-DNMT3a for 48 hrs and then analyzed by immunoblotting. The quantification of these results is shown. *p<0.05.

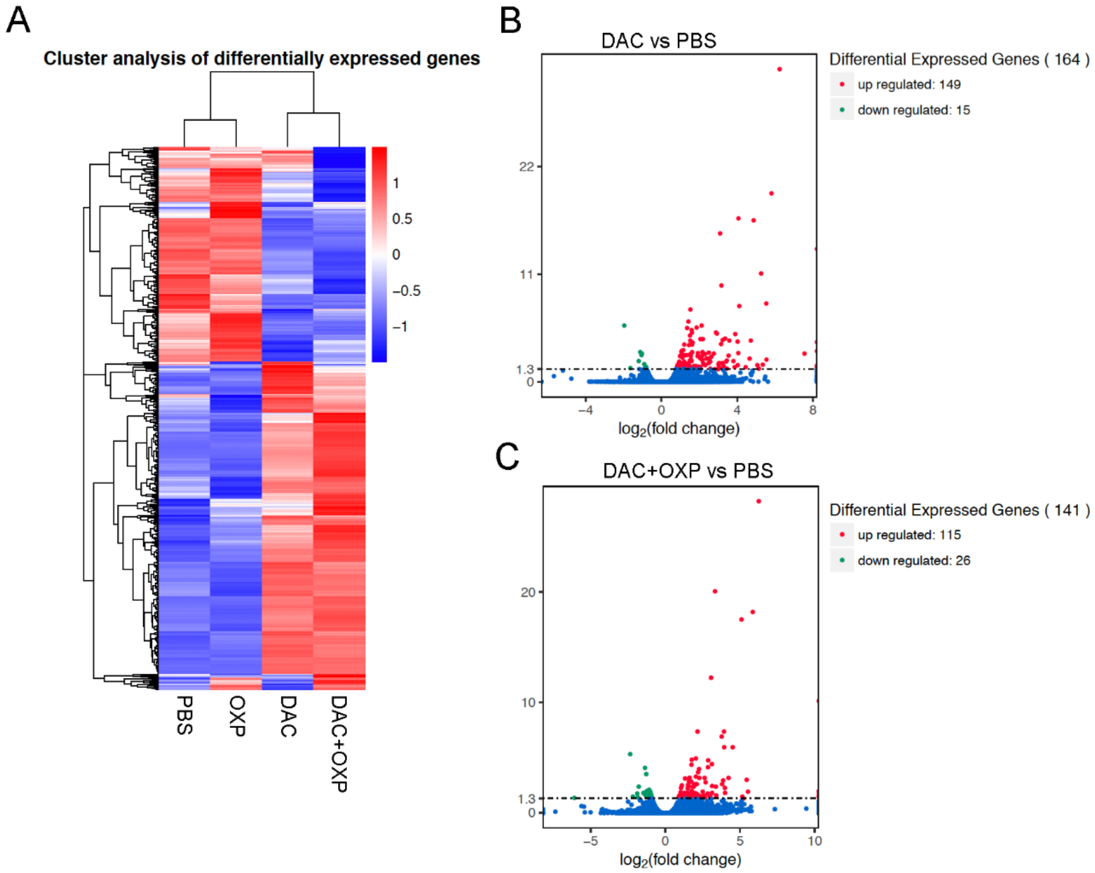

Figure S2. RNA-seq indicated that differential gene expression induced by DAC.

A. The cluster analysis of differentially expressed genes among four groups.

B. The volcano plot showed the differential expression genes between DAC and PBS group (149 upregulated genes and 15 down-regulated genes).

C. The volcano plot showed the differential expression genes between DAC+OXP and PBS group (115 upregulated genes and 26 down-regulated genes).

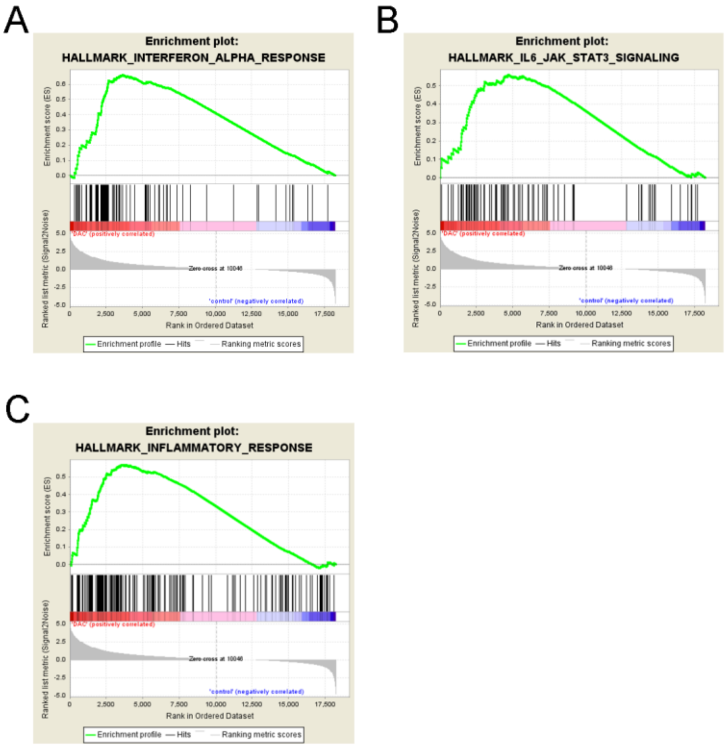

Figure S3. The GSEA plot for comparison between DAC and PBS group.

A. The GSEA plot for the signature "Interferon-alpha", which represents a set of IFN-α-related genes that exhibited upregulated expression after treatment with DAC for 5 consecutive days, is shown.

B. The GSEA plot for the signature "IL6-JAK-STAT3 signaling." after treatment with DAC for 5 consecutive days.

C. The GSEA plot for the signature "inflammatory response." after treatment with DAC for 5 consecutive days.

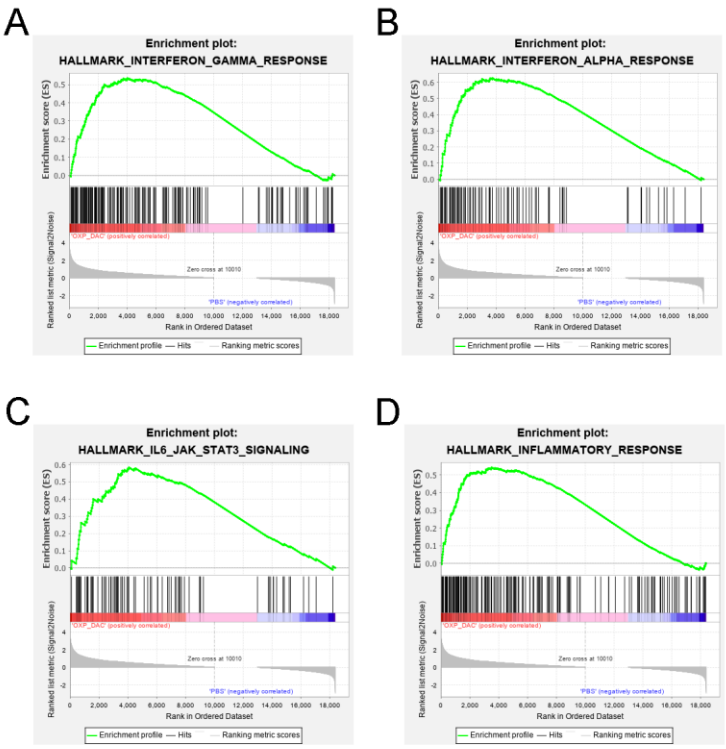

Figure S4. The GSEA plot for comparison between DAC and PBS group.

A. The GSEA plot for the signature "Interferon-gamma", which represents a set of IFN-γ-related genes that exhibited upregulated expression after treatment with DAC (consecutive days) and OXP for (2 times with 3-days intervals), is shown

B. The GSEA plot for the signature "Interferon-alpha", which represents a set of IFN-α-related genes that exhibited upregulated expression after treatment with DAC (consecutive days) and OXP for (2 times with 3-days intervals), is shown.

C. The GSEA plot for the signature "IL6-JAK-STAT3 signaling." after treatment with DAC (consecutive days) and OXP for (2 times with 3-day intervals).

D. The GSEA plot for the signature "inflammatory response." after treatment with DAC (consecutive days) and OXP for (2 times with 3-day intervals).

Figure 1A

Raw data

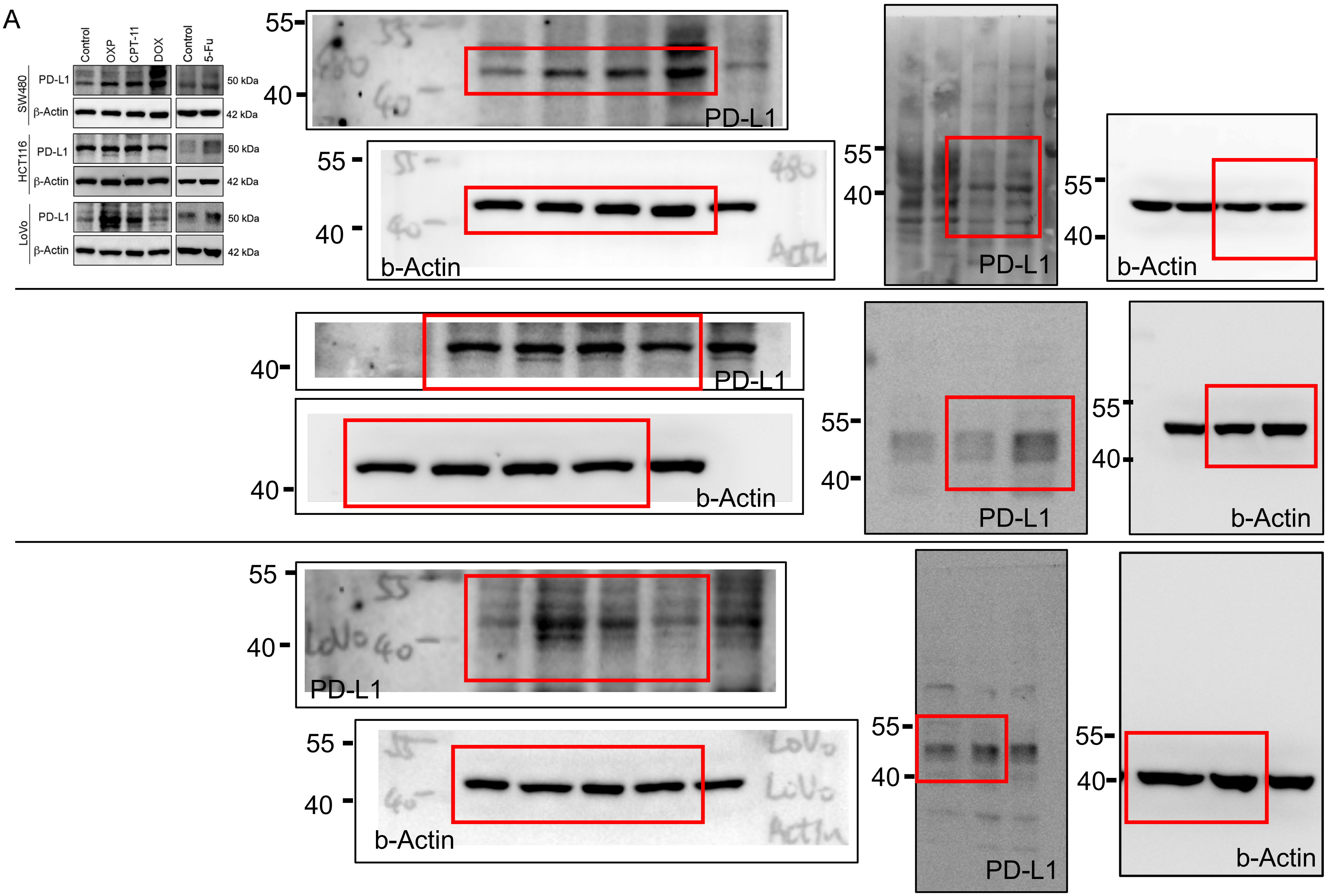

Figure 1C

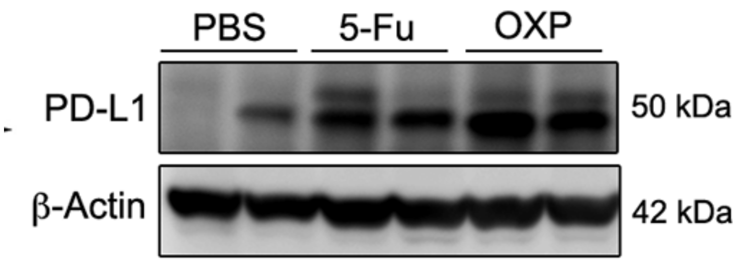
PBS
5-Fu
OXP
PD-L1
50 kDa
β-Actin
42 kDa

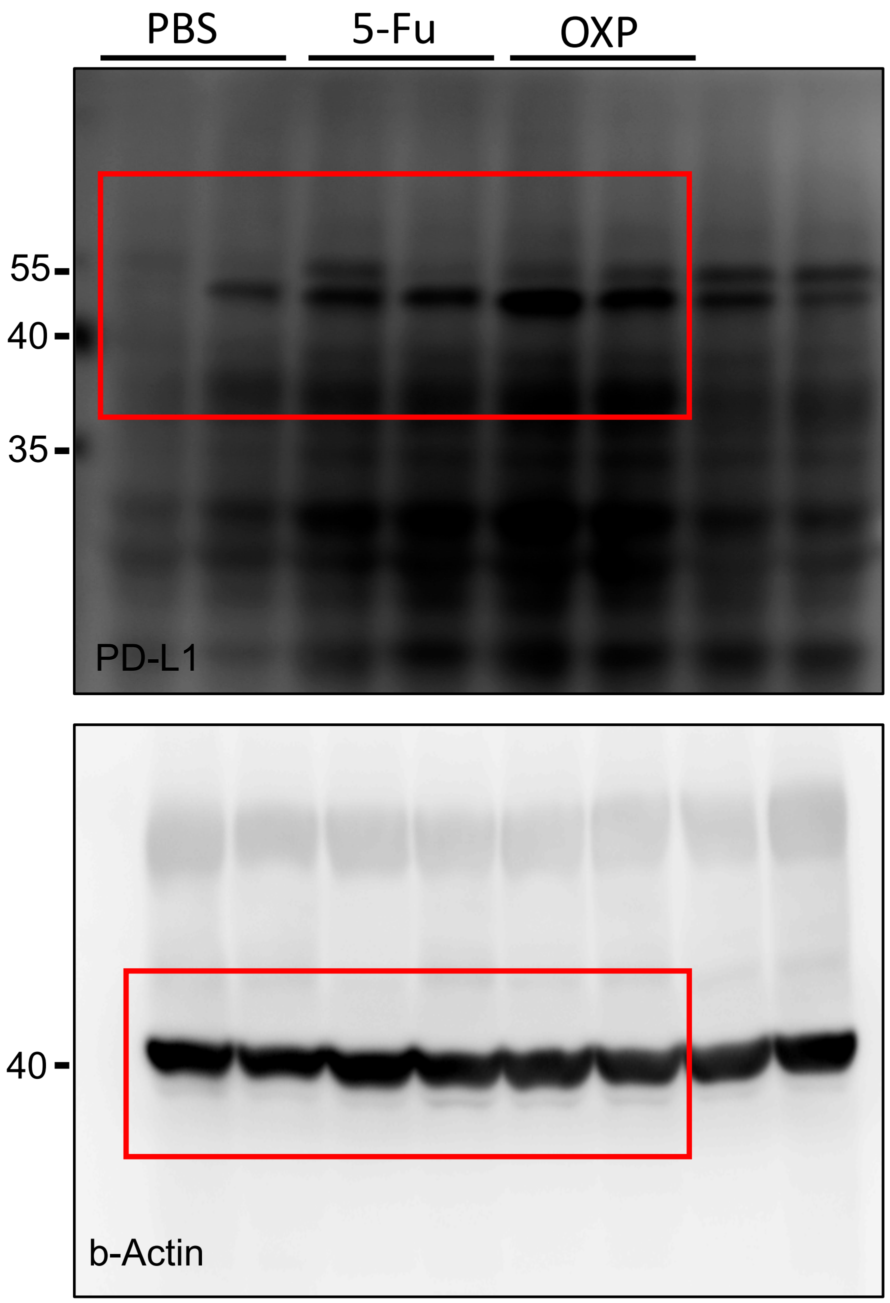
PBS
5-Fu
OXP
55
40
35
PD-L1
40
b-Actin

Figure 2A

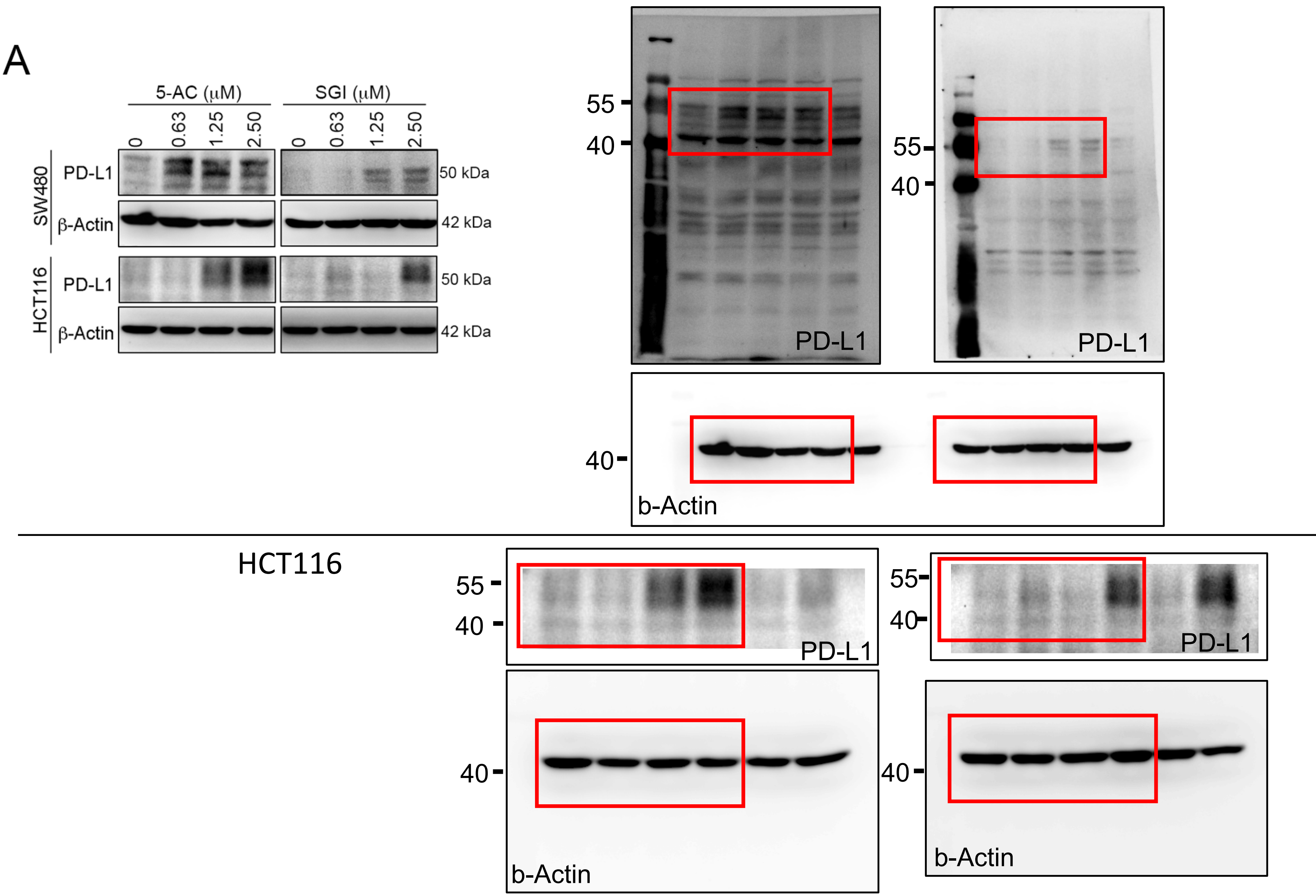

A
5-AC (μM)
SGI (μM)
0
0.63
1.25
2.50
SW480
PD-L1
β-Actin
HCT116
50 kDa
42 kDa
SW480
55
40
PD-L1
b-Actin
HCT116

Figure 2C

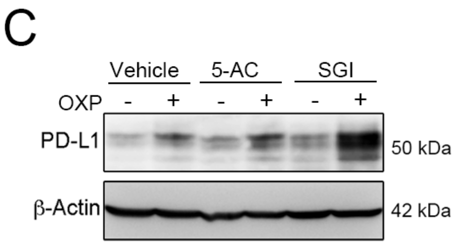
C
Vehicle
5-AC
SGI
OXP
-
+
-
+
-
+
PD-L1
50 kDa
β-Actin
42 kDa

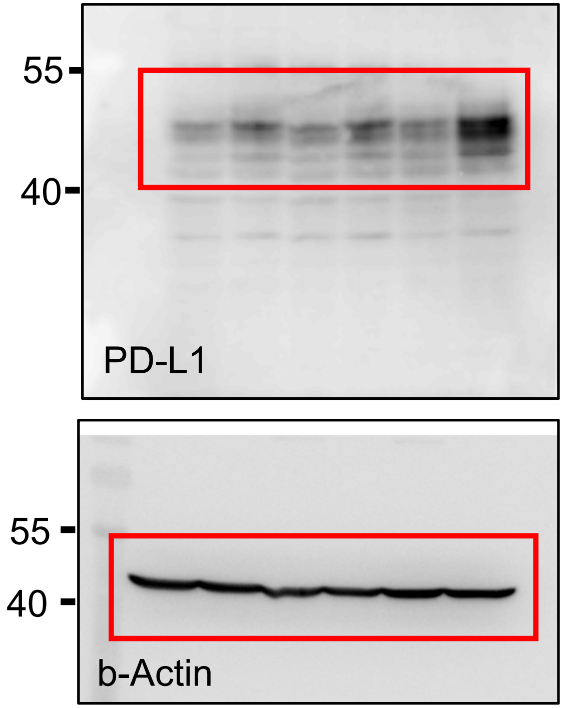
55
40
PD-L1
55
40
b-Actin

Figure 2D

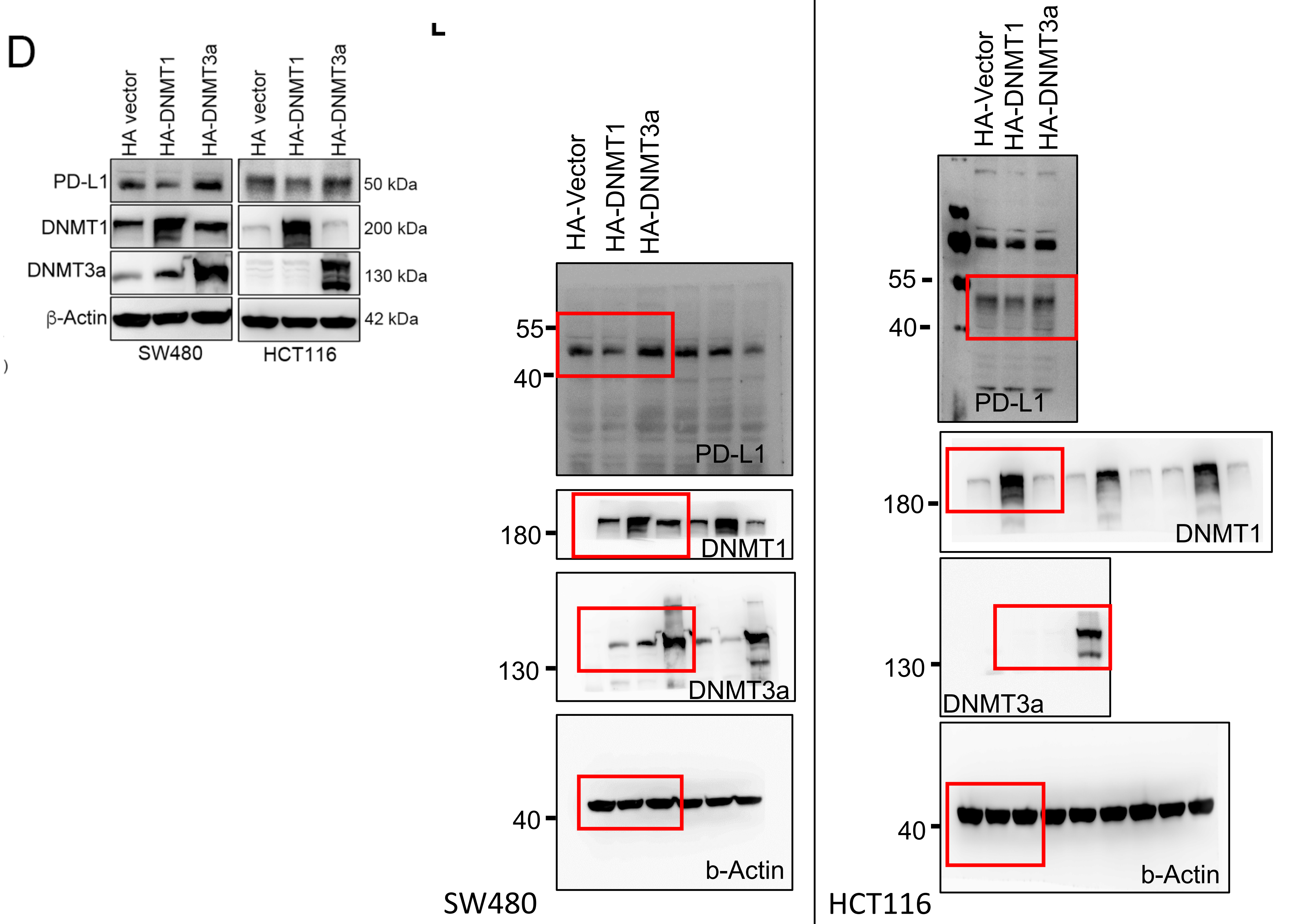

D
HA vector
HA-DNMT1
HA-DNMT3a
HA vector
HA-DNMT1
HA-DNMT3a
PD-L1
DNMT1
DNMT3a
β-Actin
50 kDa
200 kDa
130 kDa
42 kDa
SW480
HCT116
HA-Vector
HA-DNMT1
HA-DNMT3a
55
40
PD-L1
180
DNMT1
130
DNMT3a
40
b-Actin
SW480
HA-Vector
HA-DNMT1
HA-DNMT3a
55
40
PD-L1
180
DNMT1
130
DNMT3a
40
b-Actin
HCT116

Figure 2E

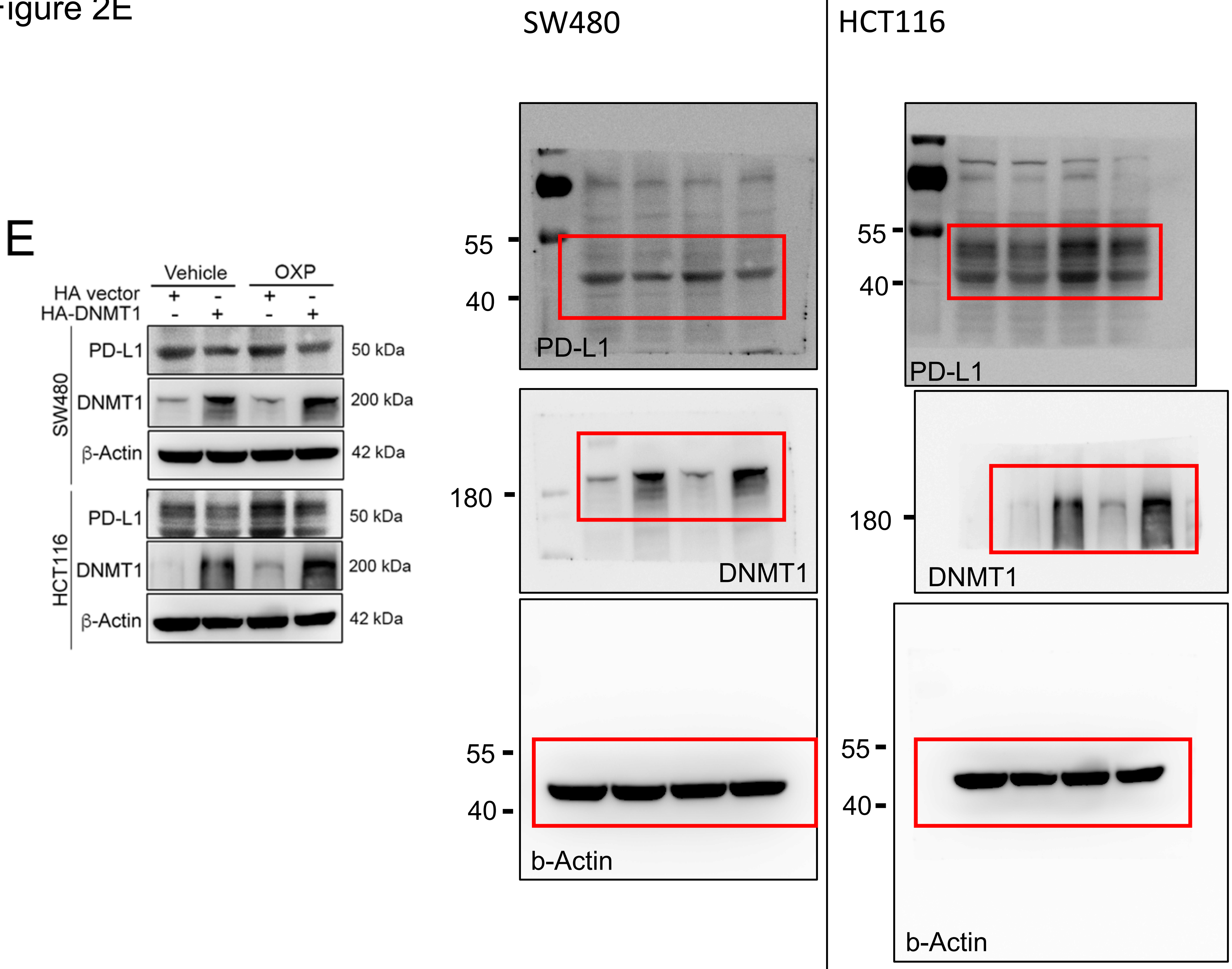
E
Vehicle
OXP
HA vector
HA-DNMT1
+ - + -
- + - +
SW480
PD-L1
DNMT1
β-Actin
50 kDa
200 kDa
42 kDa
HCT116
PD-L1
DNMT1
β-Actin
50 kDa
200 kDa
42 kDa
SW480
HCT116
55
40
PD-L1
180
DNMT1
55
40
b-Actin
55
40
PD-L1
180
DNMT1
55
40
b-Actin

Figure 2F

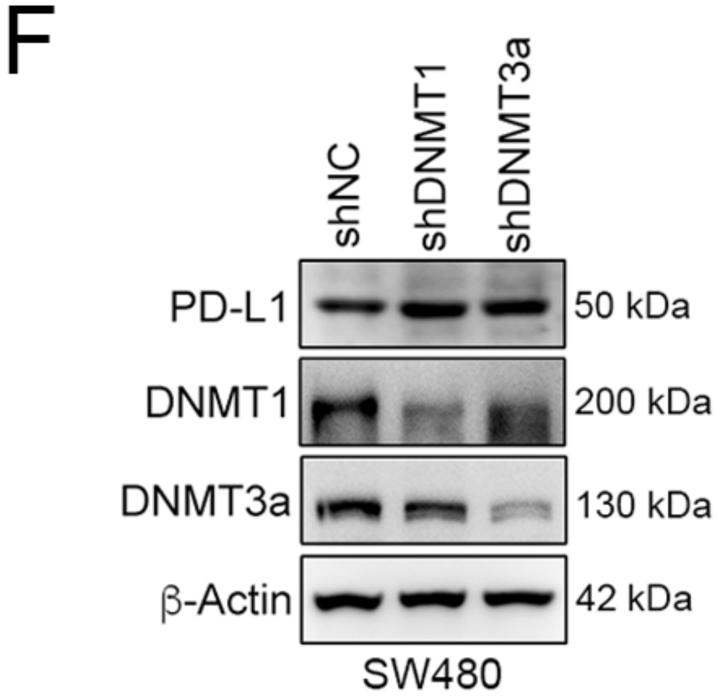

F
shNC
shDNMT1
shDNMT3a
PD-L1
50 kDa
DNMT1
200 kDa
DNMT3a
130 kDa
β-Actin
42 kDa
SW480

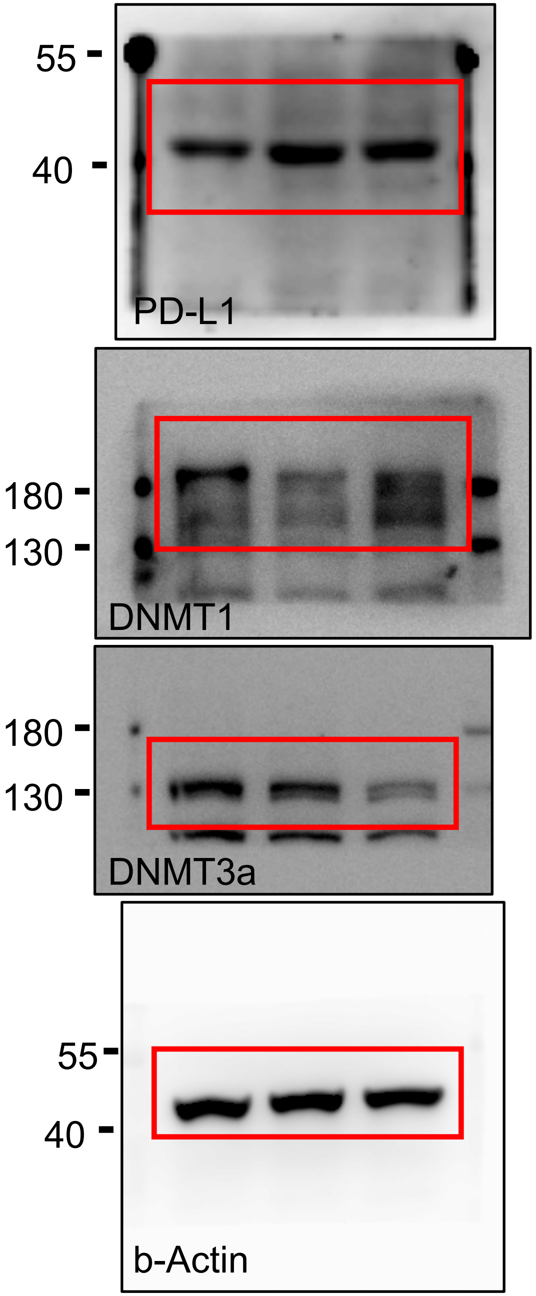

55
40
PD-L1
180
130
DNMT1
180
130
DNMT3a
55
40
b-Actin

Figure 2G

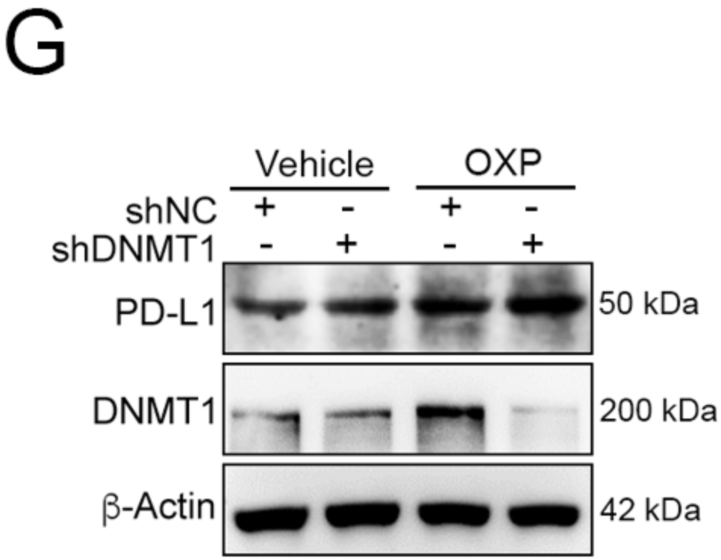
G
Vehicle
OXP
shNC + - + -
shDNMT1 - + - +
PD-L1
50 kDa
DNMT1
200 kDa
β-Actin
42 kDa

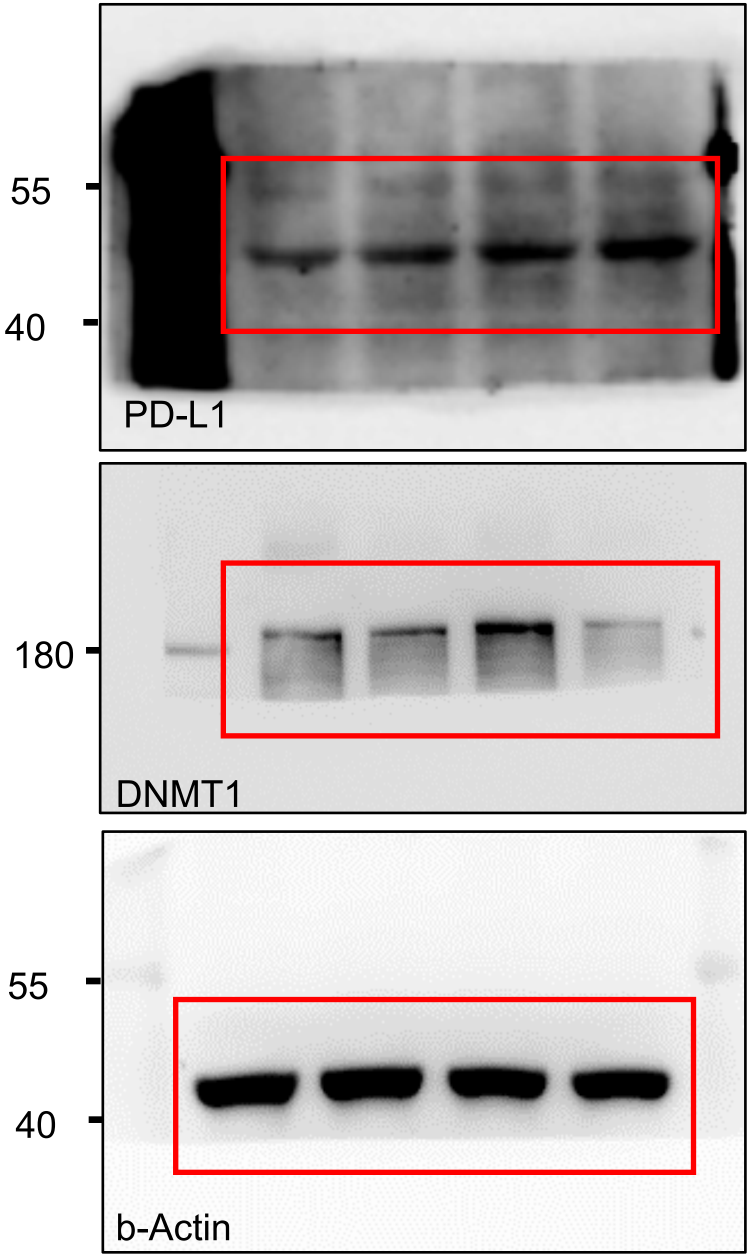
55
40
PD-L1
180
DNMT1
55
40
b-Actin

Figure 3A

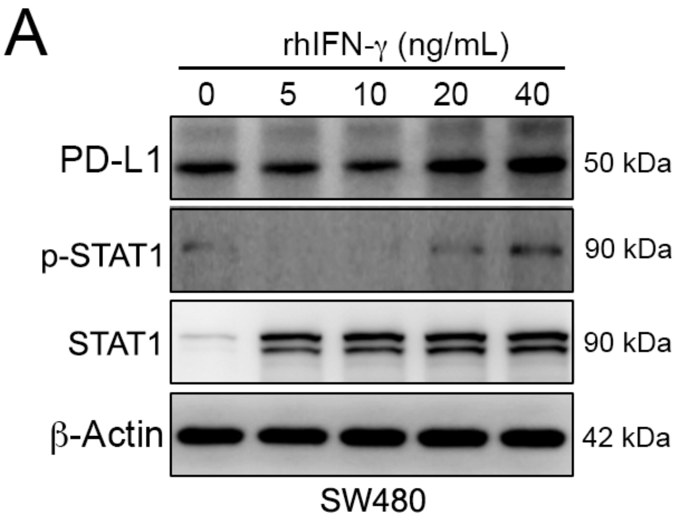
A
rhIFN-γ (ng/mL)
0
5
10
20
40
PD-L1
50 kDa
p-STAT1
90 kDa
STAT1
90 kDa
β-Actin
42 kDa
SW480

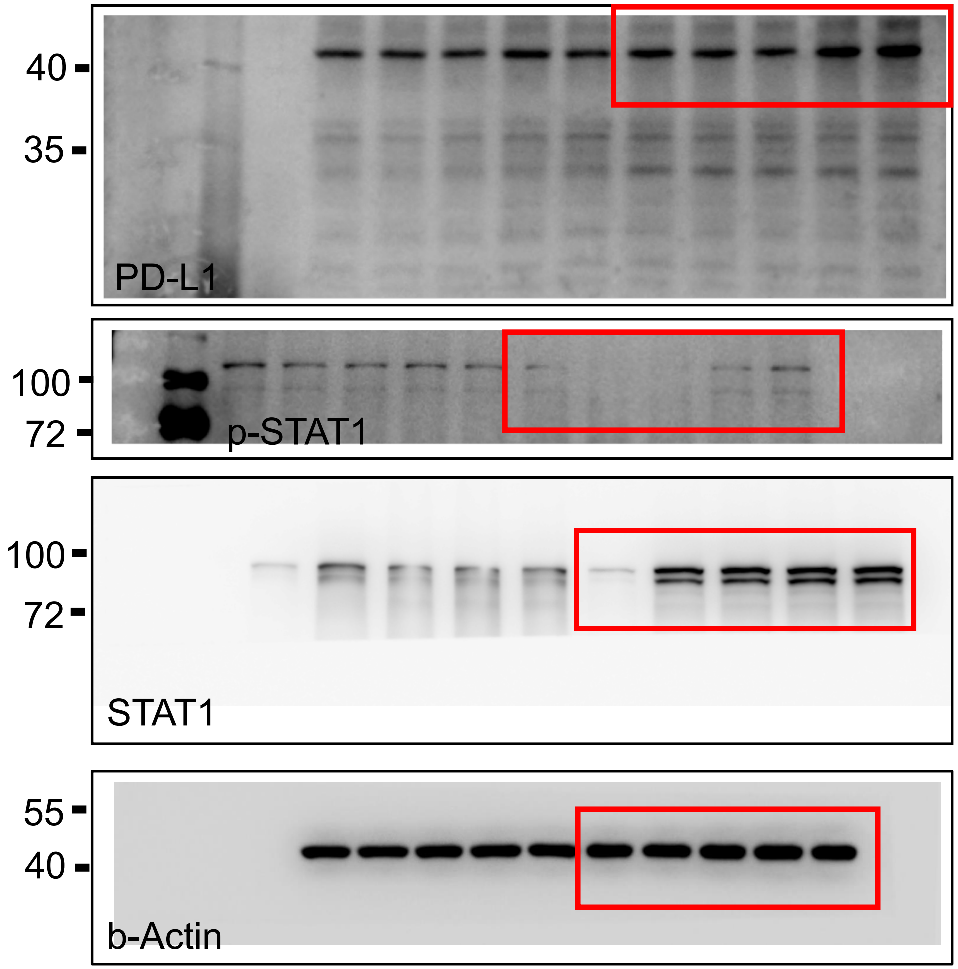
40
35
PD-L1
100
72
p-STAT1
100
72
STAT1
55
40
b-Actin

Figure 3B

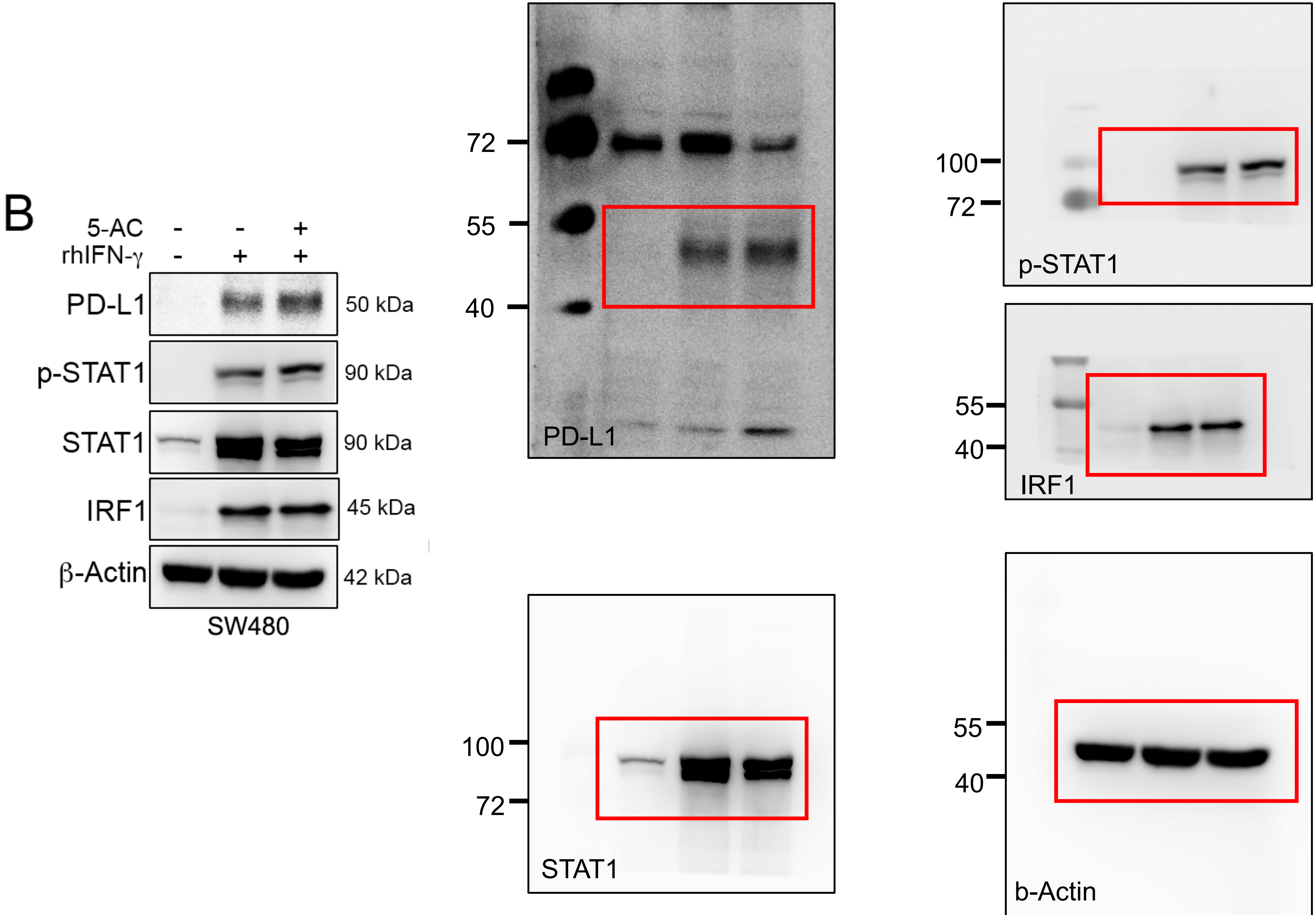
B
5-AC - - +
rhIFN-γ - + +
PD-L1
50 kDa
p-STAT1
90 kDa
STAT1
90 kDa
IRF1
45 kDa
β-Actin
42 kDa
SW480
72
55
40
PD-L1
100
72
p-STAT1
55
40
IRF1
100
72
STAT1
55
40
b-Actin

Figure 3C

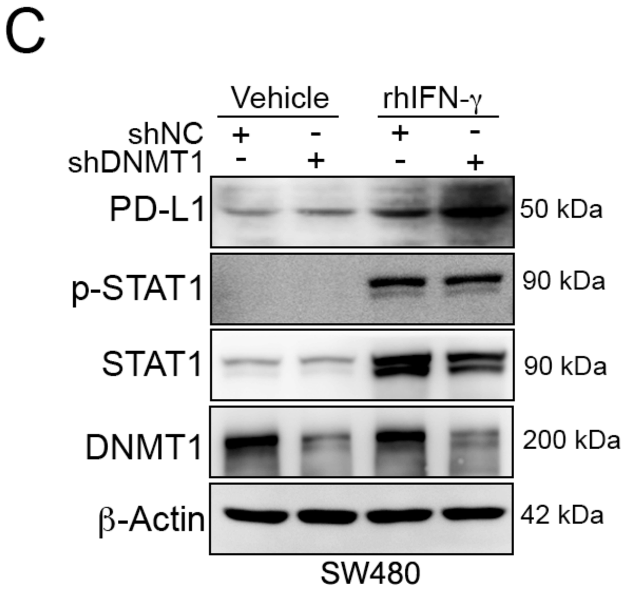
C
Vehicle
rhIFN-γ
shNC + - + -
shDNMT1 - + - +
PD-L1
50 kDa
p-STAT1
90 kDa
STAT1
90 kDa
DNMT1
200 kDa
β-Actin
42 kDa
SW480

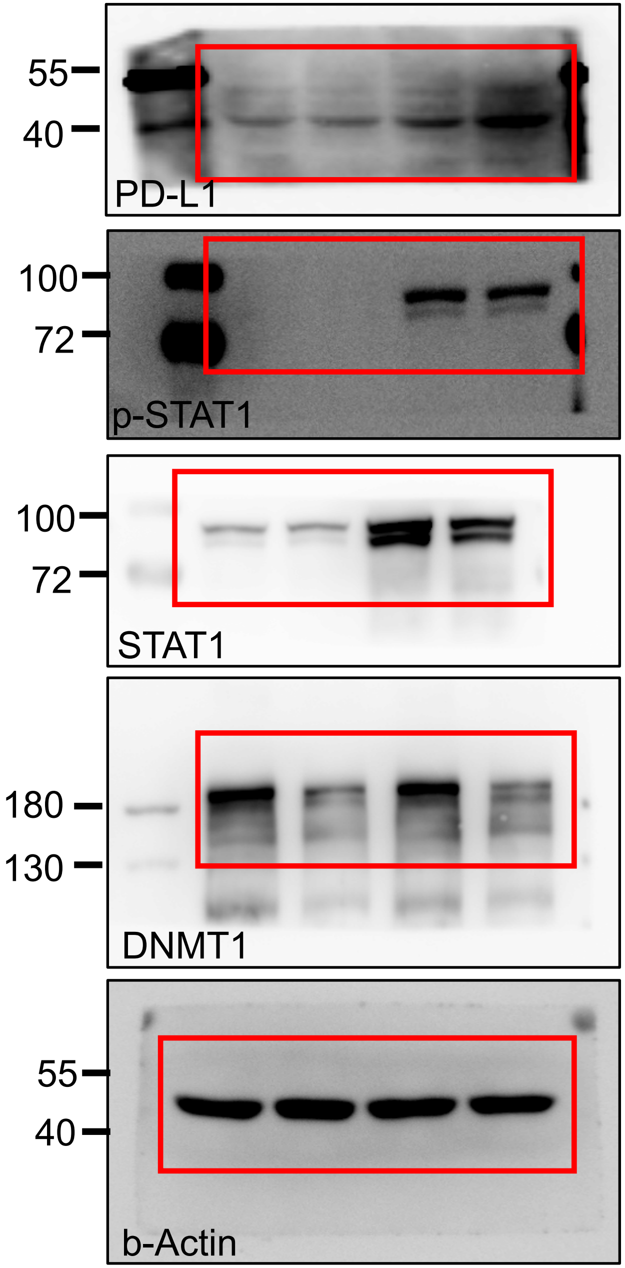
55
40
PD-L1
100
72
p-STAT1
100
72
STAT1
180
130
DNMT1
55
40
b-Actin

Figure 3D

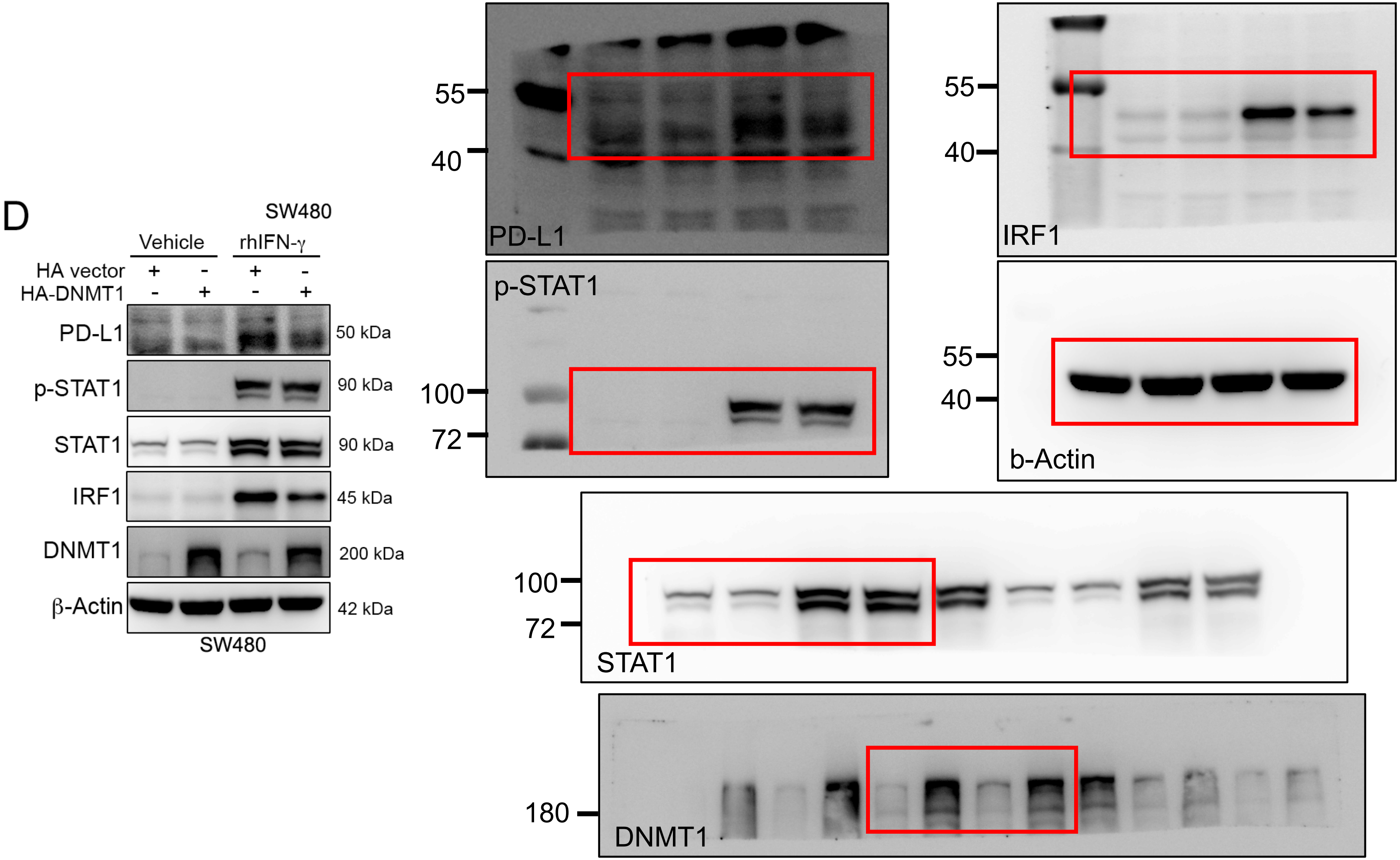

D
SW480
Vehicle
rhIFN-γ
HA vector + - + -
HA-DNMT1 - + - +
PD-L1
50 kDa
p-STAT1
90 kDa
STAT1
90 kDa
IRF1
45 kDa
DNMT1
200 kDa
β-Actin
42 kDa
SW480
55
40
PD-L1
55
40
IRF1
p-STAT1
100
72
55
40
b-Actin
100
72
STAT1
180
DNMT1

Figure 4B

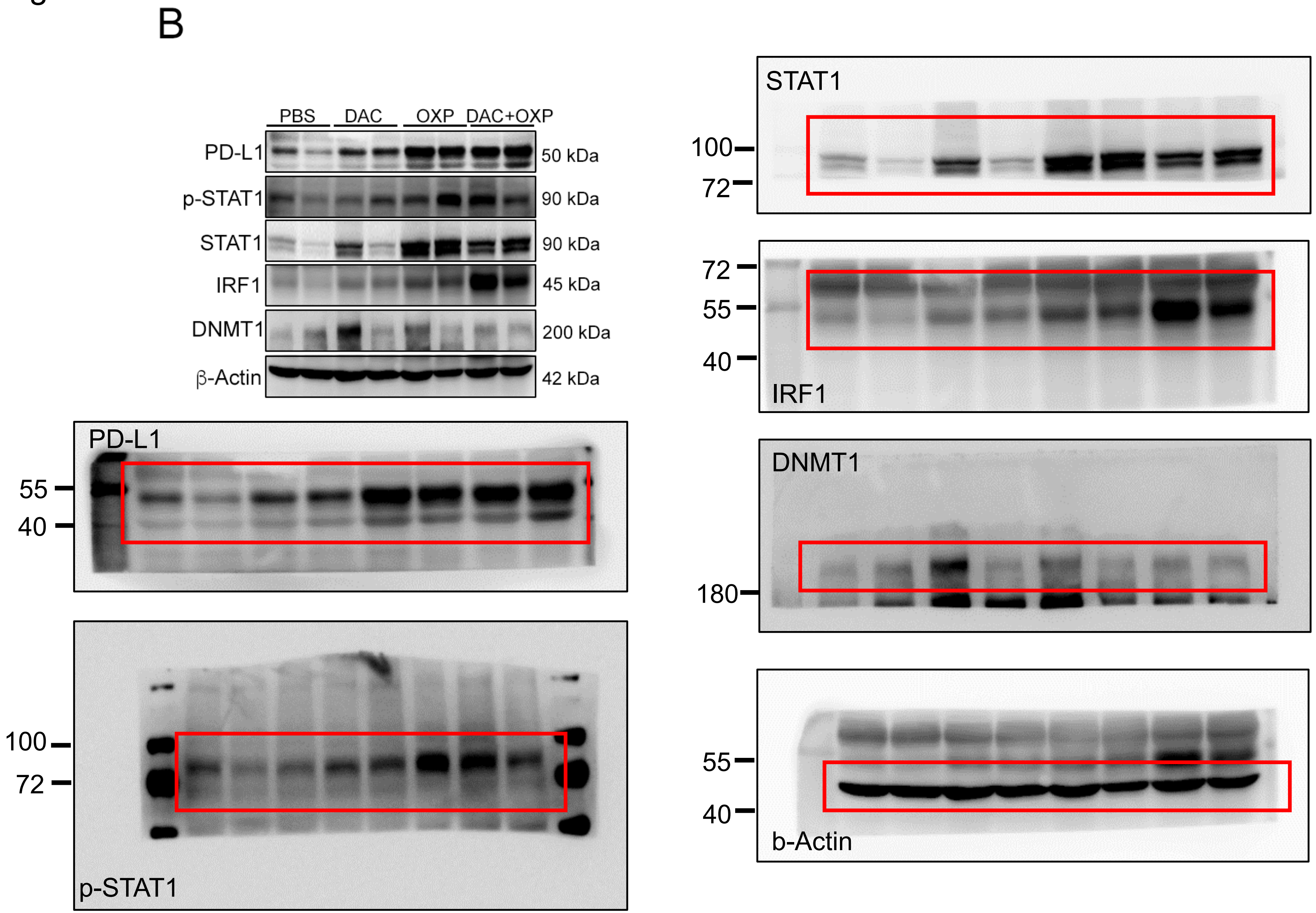
B
PBS
DAC
OXP
DAC+OXP
PD-L1
50 kDa
p-STAT1
90 kDa
STAT1
90 kDa
IRF1
45 kDa
DNMT1
200 kDa
β-Actin
42 kDa
PD-L1
55
40
p-STAT1
100
72
STAT1
100
72
IRF1
72
55
40
DNMT1
180
b-Actin
55
40
